# Identifying gene-level mechanisms of successful dispersal of Vibrio parahaemolyticus during El Niño events

**DOI:** 10.1099/mgen.0.001317

**Published:** 2024-11-08

**Authors:** Amy Marie Campbell, Ronnie G. Gavilan, Chris Hauton, Ronny van Aerle, Jaime Martinez-Urtaza

**Affiliations:** 1 School of Ocean and Earth Science, University of Southampton, National Oceanography Centre, Southampton, UK; 2 Centre for Environment, Fisheries and Aquaculture Science (CEFAS), Weymouth, UK; 3 Centro Nacional de Salud Pública, Instituto Nacional de Salud, Lima, Peru; 4 Department of Genetics and Microbiology, Autonomous University of Barcelona, Barcelona, Spain

**Keywords:** El Niño, gene association, machine learning, pathogen dispersal, *Vibrio parahaemolyticus*

## Abstract

El Niño events, the warm phase of the El Niño Southern Oscillation, facilitate the movement of warm surface waters eastwards across the Pacific Ocean. Marine organisms transported by these waters can act as biological corridors for water-borne bacteria with attachment abilities. El Niño events have been hypothesized as driving the recent emergence of *Vibrio parahaemolyticus* (Vp) variants, marine bacterium causing gastroenteritis, in South America, but the lack of a robust methodological framework limited any further exploration. Here, we introduce two new analysis approaches to explore Vp dynamics in South America, which will be central to uncovering Vp dynamics in the future. Distributed non-linear lag models found that strong El Niño events increase the relative probability of Vp detection in Peru, with a 3–4-month lag time. Machine learning found that the presence of a specific gene (*vopZ*) involved in attachment to plankton in a pandemic Vp clone in South America was temporally associated with strong El Niño events, offering a possible strategy for survival over long-range dispersal, such as that offered by El Niño events. Robust surveillance of marine pathogens and methodological development are necessary to produce resolute conclusions on the effect of El Niño events on water-borne diseases.

**Impact Statement**

*Vibrio parahaemolyticus* is a water-borne pathogen representing the most common cause of shellfish poisoning globally. In recent decades, it has undergone global expansion, including an emergence in South America; however, the mechanisms behind this are unknown. Understanding how routine environmental shifts, such as El Niño events, might drive the global expansion of water-borne bacteria, such as *V. parahaemolyticus*, is crucial to predicting future pathogen expansion. We combined both genomic and environmental data, aiming to fil these knowledge gaps by uncovering potential associations between El Niño events and *V. parahaemolyticus* in Peru. Previously only hypothesized, we quantified the lagged effects of El Niño events on * V. parahaemolyticus* detection in Peru for the first time. We also identified possible gene-level transport mechanisms based on adherence to plankton, whose dynamics are also tied to El Niño events, behind this association. Our results found that new analysis approaches, in the form of modern time series analysis and machine learning, can and should be used to uncover this uncharacterized but potentially prolific dispersal route of water-borne bacteria.

## Data Summary

The data underlying this article are available in the article and in its accompanying supplementary material. All citations and links to data from public repositories are in the Methods section. *Vibrio parahaemolyticus* isolate data reported to the Instituto Nacional de Salud can be found in Table S1. The National Center for Biotechnology Information accession numbers for sequences used can be found in Table S2.

## Introduction

Water-borne bacteria are intrinsically linked to the environment and respond dynamically to environmental shifts. *Vibrio parahaemolyticus* (Vp), a marine bacterium residing in brackish waters, is the leading cause of shellfish poisoning globally, causing at least half a million cases of gastroenteritis in humans a year [1]. The transmission of disease occurs through exposure to Vp in the environment, rather than human-to-human transmission, so understanding the presence of Vp in the environment gives us a proxy indication of Vp epidemiology, for which surveillance is limited. Environmental perturbations seen during climate oscillations can affect the ecology of these environmental Vp populations, affecting their survival, introducing evolutionary pressures and facilitating transport to new areas. Specifically, recent decades have seen the expansion of *Vibrio* clones from their endemic regions, such as the emergence and local dispersal in South America, which coincided with three significant El Niño events (*V. cholerae* associated with the 1990–1991 event and Vp associated with the 1997–1998 and 2010 events).

El Niño events, the warm phase of the El Niño Southern Oscillation (ENSO), characteristically involve the arrival of more suitable warmer conditions and the movement of warmer waters from the western to the eastern side of the Pacific Ocean, carrying a range of marine organisms and affecting the marine climate and biological community composition off the western coast of South America (Fig. S1, available in the online version of this article). It has been previously hypothesized that El Niño events could act as biogeographical corridors that allow the transport of *Vibrio* bacteria [2], with strong temporal correlations found between cholera incidence in Peru and the 1997–1998 El Niño event [3]. Environmental variability can contribute to the emergence and dispersal of marine pathogens by driving population dynamics twofold: firstly, through the introduction of non-native strains through environmental transport mechanisms such as ocean currents, and secondly, introducing environmental selection pressures that result in demographic population shifts, allowing distinct strains, with high plasticity or adaptability to the changing conditions, to emerge from and replace background populations. Horizontal gene transfer and gene expression are intrinsic mechanisms driving pathogen population dynamics by introducing adaptive advantages during particular environmental conditions.

Associations have been found between *Vibrio* bacteria and planktonic organisms that provide nutrient-rich microhabitats [4]. Though poorly understood, marine organisms carried by El Niño waters are capable of providing shelter and critical nutrients that facilitate the survival and spread of pathogenic *Vibrio* [5,6] and have been suggested to aid in dispersal and disease transmission for *V. cholerae* [7], but this has not yet been tested for Vp or in the context of El Niño events. Previous research has attempted to quantify the effects of El Niño on Vp, such as through generalized additive models [8] or wavelet geographic information system (GIS) analysis [3]. However, there is an opening for more robust methodological approaches to further explore the role of El Niño events on marine bacteria, in terms of complex, non-linear and multivariable associations that remain unknown, requiring novel analysis approaches to test such hypotheses.

Our exploratory analysis tested the potential of two modern methodologies to answer key questions surrounding the associations between El Niño events and *Vibrio* dynamics in South America, using surveillance and genomic data. Firstly, we used distributed non-linear lagged time series analysis to assess our ability to quantify complex lagged associations between El Niño and Vp dynamics. These are commonly applied to environmental epidemiology problems and have recently been used to explore *Vibrio* outbreaks in the USA [9] but have not yet been applied to this specific context in South America or for Vp specifically. We then deployed machine learning models to predict accessory gene presence based on environmental and ecological drivers to explore possible gene-level variations in response to El Niño events. Machine learning has been used recently to detect the probability of Vp abundance due to environmental conditions [10,11] but has not been utilized in the context of understanding complex environmental responses, including selective pressures and adaptation, at the gene level. There is a pressing need to utilize these novel methods in this context to provide new insights into complex mechanisms not characterizable by previous traditional association methods. Understanding these dynamics will be critical for forecasting future health outcomes of the current strong El Niño event in 2023–2004 [12] or further climate phenomena associated with the emergence of climate-sensitive pathogens.

## Methods

### Time-series analysis

Vp infections are classically underreported, so similar to Martinez-Urtaza *et al*. [8], we used a proxy to represent Vp populations in Peru, as continuous water sampling for environmental Vp would be infeasible. We used the number of isolates reported to the Instituto Nacional de Salud in Lima, Peru, between 1994 and 2017 from hospitals and public health laboratories across the country (Table S1). Here, similar to most regions, there is no routine Vp surveillance; however, reporting of *V. cholerae* has been mandatory since an epidemic in 1991, leading to parallel identification of other *Vibrio* bacteria. This provided an inferred infection rate, however likely underestimates Vp incidence as most infections are mild and do not seek out medical assistance, which leads to such clinical isolates. A similar methodology of inferred infection rates was used by Martinez-Urtaza *et al*. [8]. This collection of isolate counts was conducted within the framework of the National Surveillance for Acute Enteric Diarrhea approved by the Instituto Nacional de Salud of Peru, and the Committee of Research and Ethics approval was waived in accordance with the national legislation and the institutional requirements for Public Health Surveillance (Ministry Resolution N.° 730-2022-MINSA). All isolate data have been anonymized. The final total of 639 anonymized isolate counts can be found in Table S1.

The metric used to quantify ENSO phases was the multivariate ENSO index (MEI) v2 [13]. This bi-monthly time series is calculated as a function of variables [sea-level pressure, sea surface temperature (SST), surface wind and outgoing longwave radiation] over the tropical Pacific basin. Warm periods (El Niño events) are quantified using a threshold of over 0.5 and cold periods (La Niña events) similarly for under −0.5. These data are available from 1979 to 2024 at https://psl.noaa.gov/enso/mei/. To extract the effect of El Niño events amidst seasonal and demographic signals, we acquired population time series data from the United Nations World Population Prospects data [14] and temperature data from the World Bank Climate Change Knowledge Portal [15] for the period.

To quantify the impact of El Niño effects on relative risk, in this case represented by the number of Vp isolates reported, we used the following packages in R [16]: dlnm v2.4.7 [17], tsModel v0.6, splines v3.6.2, mgcv v1.9 [18], Epi v2.47.1 [19] and lubridate v1.9.3 [20]. The relative risk here refers to the number of reported (Vp isolates in Peru from 1994 to 2017 as a proxy for the number of Vp infections in Peru (Table S1).

We first fitted a Poisson regression model assuming no lags. We removed the effects of seasonality using a natural spline function to represent a hot and cold season (2 degrees of freedom for each of the 24 years) and included a function representing the day of the year. We also incorporated functions to account for seasonal temperature change and population changes throughout this period, using population time-series data from United Nations World Population Prospects data [14]. We then modelled the bidimensional exposure–lag–response association between El Niño phases and relative Vp risk by combining two functions defined within a cross-basis term, as per the distributed non-linear model methodology set out by Gasparrini [21]. We tested a range of parameterizations of the cross-basis and chose the one with the lowest Akaike information criterion value – a relative measure of statistical model quality. Specifically, we selected a natural spline function with two internal knots at equally spaced log values over 6 months of lag to model the lag–response dimension and a natural spline function with five equally spaced knots defined by a quadratic b-spline over the MEI distribution to model the exposure–response curve. The resulting set of coefficients were used to model the bidimensional exposure–response–lag landscape.

Interrupted time-series analysis was used to show the influence of the 1997/1998 El Niño event on the number of Vp isolates recovered, using the methodology set out by Bernal *et al*. [22]. We created a subset of our data between 1996 and 1998 to specifically focus on this one event. We implemented a step-change model, using a binary code to indicate when the ENSO was in a warm phase (*n*=1) compared to a counterfactual where there was no warm phase (*n*=0 throughout). Confounding effects from seasonality (with higher Vp presence expected in summer months) or long-term trends (as surveillance or awareness increases) were controlled by using a dummy variable to represent temperature and a linear term representing time.

### Genomic analysis

*Vibrio parahaemolyticus* sequence type 3 (VpST3) strains were selected from the global, publicly accessible National Center for Biotechnology Information database, selecting strains that were accompanied with sufficient metadata (minimum of a year and country). Sixty-six South American strains were used for gene association analysis, 51 from Peru, 8 from Chile and 7 from Colombia, isolated over a period of 1997–2019. Accession numbers for each sequence can be found in Table S2. Bactopia v2.0.2 [23] was used for processing, validating, quality filtering, assembling and annotating the raw VpST3 sequences (Table S2). We identified SNPs in the core genome using Parsnp v1.5.6 [24] and created a core genome alignment from these using the reference genome Vp RIMD 2210633.

Population dynamics of the VpST3 isolates were explored using a Bayesian phylogeographic approach in BEAST v2 [25]. Effective population size for the VpST3 populations in South America over time was reconstructed using a Coalescent Bayesian Skyline tree prior, utilizing a Jukes-Cantor (JC69) site model with a strict molecular clock, in the Markov chain Monte Carlo (MCMC) sampling procedure until convergence was reached, with a final total of 100 000 000 generations. The convergence of the MCMC outputs (effective sample size >200) was confirmed using Tracer v1.7.1 [26], which then provided summary statistics and was used to produce Bayesian Skyline plots. Roary v3.13.0 [27] was used to construct the pangenome and annotate the presence of genes within the VpST3 isolates. A global phylogeny using these strains amidst a total of 312 global strains, previously computed by Campbell *et al.* [28], was used to assess lineage dependence of the genes of interest.

### Machine learning

We identified accessory genes present in 20–90% of the South American strains based on two key criteria: firstly, these genes were outside of the core conserved genes but with sufficient maintenance across the population to suggest a spatiotemporally variable benefit linked to biological strategies for survival or dispersal under particular environmental conditions; secondly, this provided a sufficient number of instances of gene presence and absence for machine learning model development. From this, 15 named accessory genes were used to explore how their presence was associated with environmental variables and particularly El Niño events. We averaged the presence of each gene for each year in the time series, across all isolates found in that year, to form the dependent variable being predicted.

This temporal data frame was then appended with annual averages of the various environmental metrics. Alongside the MEI, data were collected for other drivers that could drive dynamics, including environmental metrics, spanning this period. For the period 1996–2021, global SST anomaly data were acquired from the Extended Reconstructed SST National Oceanic and Atmospheric Administration (NOAA) dataset [29], sea level change from altimetry provided by the NOAA Laboratory for Satellite Altimetry [30] and global mean temperatures from the Global Historical Climatology Network (GHCN) version 3 [31]. Local (Peruvian coast) SST data were acquired for ERA5 reanalysis – the fifth-generation European Centre for Medium-Range Weather Forecasts atmospheric reanalysis – which uses the Hadley Centre Sea Ice and Sea Surface Temperature 2 dataset pre-2007 [32] and local salinity data from Met Office Hadley Centre EN4.2.2 quality-controlled ocean data [33,34] for reconstruction of effects of El Niño events in the local environment. Shellfish movement data were also extracted from the Food and Agriculture Organization of the United Nations FishStatJ database, for both exports and imports, measured in tonnes of net product weight [35]. The shellfish trade has previously been hypothesized as an alternative transmission route for the spread of Vp [36] – specifically global movements alongside exports from Asia (endemic Vibrio region) and imports into South America. SST data were later removed due to multi-collinear effects, as it was significantly correlated (*P* < 0.01) with the El Niño (MEI) index.

The data frame provided inputs to a random forest classifier model, included in the scikit-learn v1.3.0 package [37], which is a suitable ensemble machine learning method for binary inputs (such as presence and absence data). The synthetic minority oversampling technique was used to generate more instances of the minority class (whether that was gene presence or gene absence) based on k-nearest neighbours [38], which were then randomly split into a training dataset (containing 70% of the data) and an unseen test dataset with the remaining 30%. For each gene of interest, a random forest classifier with 50 estimators was trained and tested to return binary predictions from the unseen driver data – predicting the gene to be present (1) or absent (0) based on the provided driver data.

One particular gene provided the focus for the in-depth gene-association analysis, *vopZ*, due to finding significant (<0.05) associations with the MEI and its functional role in attachment that was pertinent to the study hypothesis. Before focusing on *vopZ*, we assessed whether the pattern of *vopZ* presence was a consequence of population structure by exploring its appearance in the phylogeny or if it could be a component of an eco-evolutionary response to El Niño events. The transparency of this specific machine learning method allows us to interrogate such predictions and their associations with the datasets provided. The contribution of each driver (including the El Niño index) was investigated by interrogating the model’s feature importance values (based on Gini importance, which is the total decrease in node impurity provided by the feature) and calculating permutation importance (evaluating the contribution of each driver by replacing it with random values and observing the decline in model effectiveness).

## Results and discussion

### Non-linear lagged association analysis

The modern time-series analysis methods we applied could quantify complex, lagged temporal associations between El Niño events and the epidemiology of Vp in South America. While the 24-year time period studied (1994–2017) included the emergence of a particular Vp strain in South America in 1997, called sequence type 3 (VpST3), we first explored the general effects of El Niño on all Vp populations over time.

**Fig. 1.**
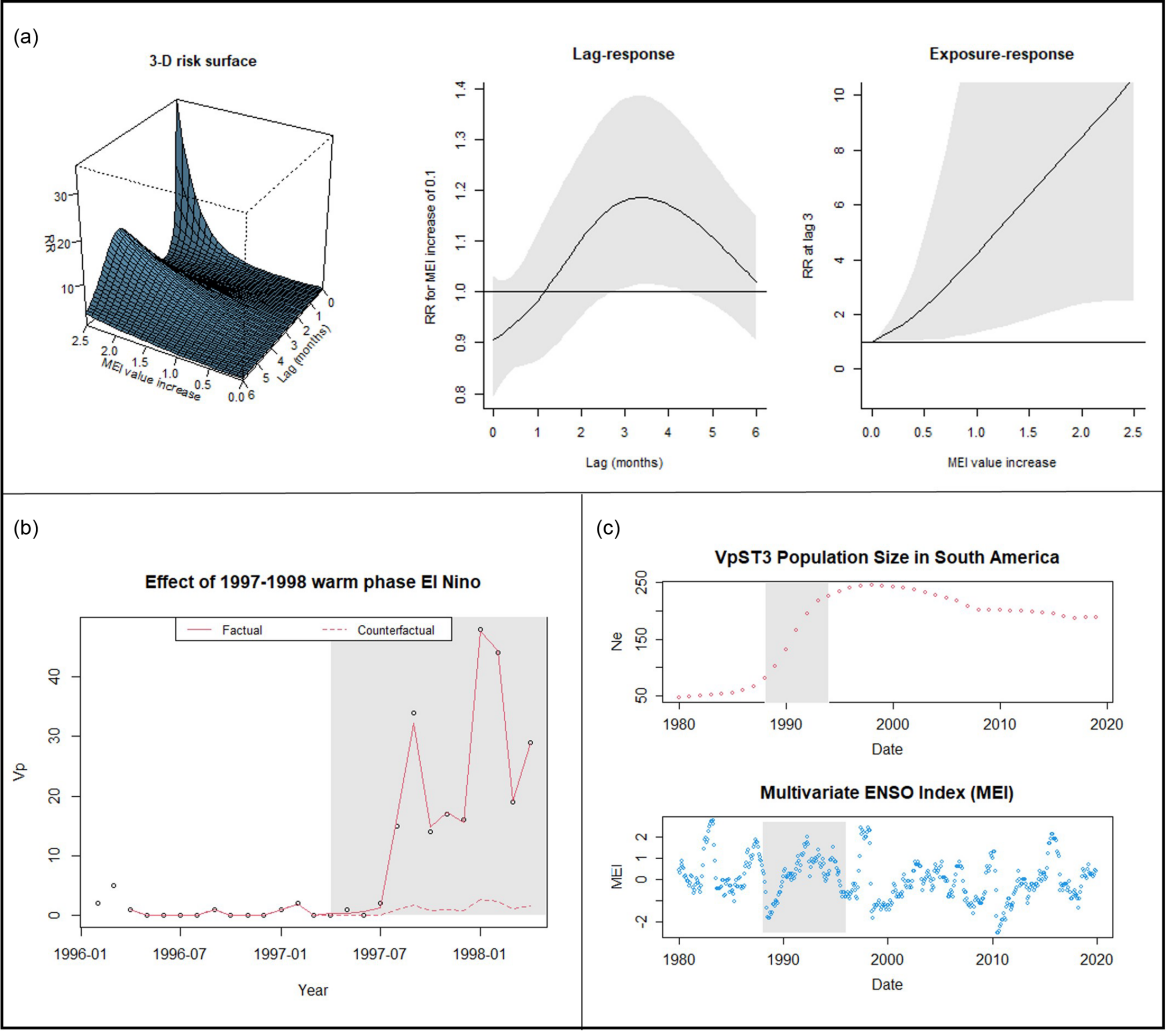
Exploring the potential of modern time-series analysis methods to quantify the associations between Vp dynamics and El Niño events. (**a**) Distributed non-linear lag model of relative risk (RR) association between MEI and increases in Vp detection, with the 3D risk landscape, the lag–response curve of Vp detection risk with a 0.1 MEI increase and the exposure–response curve for a range of MEI value increases for a specific 3-month lag. (**b**) Interrupted time-series regression showing the influence of the 1997/1998 El Niño event on the number of Vp isolates recovered compared to a counterfactual based on usual conditions. (**c**) Comparative time series of effective population size (Ne) of pandemic Vp variant ST3 in South America against MEI, with shaded region indicative of the rapid VpST3 population expansion period observed from 1988 to 1995.

Initially assuming no lags, the generalized linear model found an increase of 0.1 in the El Niño index to increase relative risk (number of Vp isolates reported) by 0.9% and an increase of 0.5 in the El Niño index to increase relative risk by 4.8% (Fig. S2). Within a distributed non-linear lag model [21], we found there is a lagged effect on Vp risk, with the relative risk increase peaking at around 3–4 months after the increase in El Niño index (Fig. 1a). Specifically, after an El Niño index increase of 0.1, we observed an 18.0% increase in relative risk (95% confidence interval: 0.8–37.9%) 3 months after and a 17.2% increase in relative risk (95% confidence interval: 1.1–35.9%) 4 months after (Fig. 1a).

Focusing on a specific El Niño event, the notable warm phase in 1997–1998 that coincided with increases in Vp isolates recovered and the emergence of a new strain, we deployed interrupted time-series regression analysis [22]. We found that the 1997–1998 El Niño event was associated with a higher number of recovered Vp isolates than would be expected under normal conditions, e.g. business-as-usual seasonal temperatures and population change (Fig. 1b).

Having found a relationship between El Niño events and Vp populations in general, we then focused deeper to explore whether this analysis could identify whether El Niño events contributed to the emergence of the particular effective pandemic VpST3 clone. Using genomic sequence data of VpST3 (Table S3), we reconstructed a time series of its effective population size during its emergence in South America. Looking beyond general census increases, effective population size gives an indication of the effects of El Niño events on variation within the population. During the rapid population expansion observed from 1988 to 1995, we find a significant positive association between effective population size and the El Niño index (*R*=0.81, *P*<0.05), with a further stronger association identified with the MEI of the previous year (*R*=0.97, *P*<0.01); however, no association was found after the initial rapid increase of variation (Fig. 1c). This indicates that if El Niño events have a role in increasing variation in Vp populations, by encouraging the addition and mixing of new populations, this is related to the expansion of Vp to new areas, with less of an effect once a specific population has become established post-introduction.

This modern time-series analysis particularly would be improved with greater spatiotemporal resolution surveillance data of Vp (epidemiological, genomic or environmental) to fully interrogate possible associations between environmental variability driven by El Niño in the marine environment.

### Machine learning for gene-level association analysis

This identification of temporal associations between Vp isolate recovery and El Niño events led to the testing of a second methodology to explore gene-level mechanisms that could characterize the functional eco-evolutionary relationship behind this association. One of the functionally annotated accessory genes – *vopZ*, a type III secretion system (T3SS) effector – emerged from our analysis as its presence appeared to be associated with the El Niño MEI, providing an exemplar gene to test our hypotheses. While other T3SS pathway effectors – *vopA*, *vopC*, *vopL* and *vopT* [39] – were core genes (present in >90% of South American strains), this specific effector was present in 84% of strains, suggesting that it provides enough benefit for uptake and maintenance that it has not been eroded away by genetic drift but potentially under specific conditions as it is not within the core conserved genes.

**Fig. 2.**
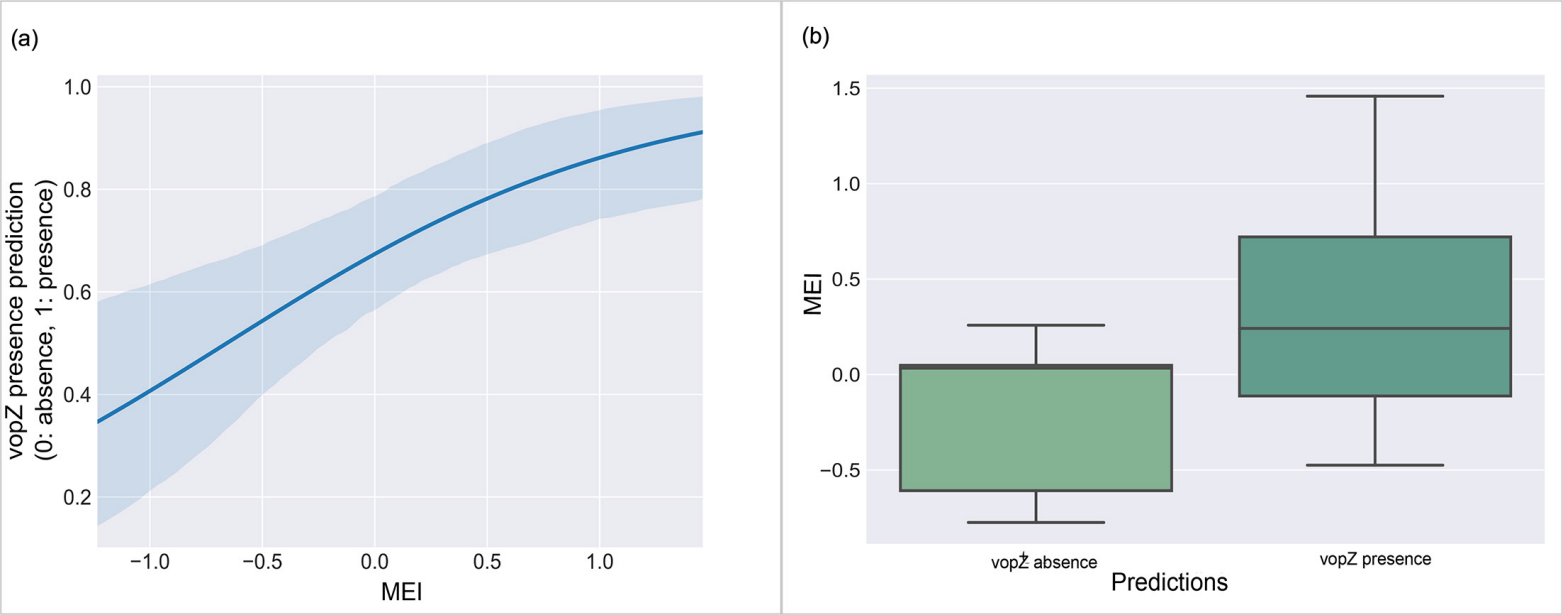
Focus on *vopZ* gene presence in South American VpST3 isolates associated with El Niño index to demonstrate machine learning potential. (**a**) Logistical regression (68% confidence interval) between El Niño MEI and forecasted *vopZ* presence in South America when random forest model was applied to unseen isolates. (**b**) Binary prediction ranges of *vopZ* presence against El Niño index when random forest model was applied to unseen isolates.

While we found no evidence of a temporal uptake trend (Fig. S3), or any inherent population structure defining the gene’s presence throughout the phylogeny (Fig. S4), suggesting that this was not a lineage-specific gene, we did find that its presence could be predicted using eco-evolutionary drivers in our machine learning analysis. The total accuracy for the model predicting *vopZ* presence (taking into account all true positives, true negatives, false positives and false negatives) was 0.778, with an F1 score (a harmonic mean of precision and sensitivity) of 0.8 and an area under the curve (of the receiver operating curve) score of 0.833, providing support for the drivers as predictors of *vopZ* presence. We found that the model predictions of *vopZ* presence were positively correlated with the El Niño index (Fig. 2a), with the interquartile prediction range for *vopZ* presence existing entirely in the positive El Niño index range (Fig. 2b). Amidst the range of drivers, the El Niño MEI emerged as the strongest predictor for *vopZ* presence, based on interrogated Gini feature importance and permutation importance (Fig. S5).

Placing the results of this method application into context, *vopZ* plays a distinct functional role in the T3SS pathway by enabling intestinal colonization [40,41]. Studies have also found that *vopZ* promotes interaction with marine hosts by binding to chitinous exoskeletons, such as plankton, and facilitating invasions into plankton communities [41,42]. Though the mechanisms of *vopZ* remain less known, similar proteins offering intestinal attachment mechanisms in humans have been found to be functionally reapplied to attach also to chitinaceous surfaces of planktonic organisms within the marine environment, such as the *GbpA* protein in *V. cholerae* [43,44], highlighting how human host colonization and marine organism attachment are intrinsically connected. This planktonic attachment enhances environmental survival [44] but can also act as an effective dispersal mechanism for marine bacteria, in which the pathogen can hitchhike on migrating zooplankton [45], facilitated by adherence proteins found in pathogens [46], such as these. A known typical consequence of El Niño events off the western Peruvian coast is large species composition changes in plankton communities due to reductions in upwelling and primary productivity [47,48], resulting in declines of native zooplankton species and introductions of foreign equatorial zooplankton species, with a shift towards smaller copepod species [8,49, 50].

We therefore note the presence of a gene offering plankton attachment opportunities that is more likely to be present during El Niño conditions in an area where this results in strong climate signals. Having previously identified VpST3’s observed dispersal in South America aligned with positive El Niño events, it is possible that populations with this gene mechanism could have opportunistically taken advantage of the unique plankton movements during the events to replace less well-adapted local populations, where, because this T3SS effector codes for a virulence mechanism, they were then able to cause outbreaks. The lack of a temporal uptake trend of *vopZ* presence could suggest shifting priorities, wherein vopZ acquisition only proved beneficial during particular conditions, with uptake potentially more favourable during prolonged adverse conditions found during long-range dispersal compared to established locally adapted populations where the benefit of dispersal abilities decreases [51]. The presence of this gene is not specific to South American samples, with possible gene enrichment in various regions where selective pressure favouring long dispersal survival exists. Long-range dispersal is not a feature exclusively associated with El Niño events but instead a basic mechanism facilitating the global epidemic dynamics of any *Vibrio* bacteria or marine pathogen reaching and colonizing new regions [52]. These patterns of *vopZ* presence start to offer insights into the eco-evolutionary role of El Niño events, as an example dispersal mechanism, in water-borne disease transmission, providing insight into survival mechanisms for dispersal in the form of dynamic planktonic host relationships. Forming more robust conclusions in the future requires higher genomic surveillance across a range of ENSO cycles, gene expression studies to characterize how VpST3 responds to changing plankton communities and dispersion models accompanied by *in situ* sampling to identify dispersal patterns of plankton-attached marine bacteria.

## Conclusions

Our exploratory analyses have highlighted the potential of state-of-the-art methodologies in generating novel insights into the emergence of water-borne infections amidst complex climatic conditions. Specifically here, they provided insight into the associations between El Niño events and Vp dynamics; however, such techniques could be applied to explore how various climate phenomena mediate the emergence and evolution of a range of pathogens and particular variants. Importantly, these analysis methods are limited by the genomic surveillance inputs – with only 66 genomes of sufficient metadata and quality available for the machine learning gene association analysis. This study utilized all available data to make inferences for the unique opportunity to explore the consequences of the strong signal of El Niño events in South America; however, robust surveillance data would greatly improve prediction potential and the strength of any associations explored here. The Peruvian government implemented their active surveillance system for *Vibrio* diseases as a response to the emergence of cholera in 1991 and subsequent reemergence in 1998, which allowed the successful detection of a global emerging strain VpST36 [53], highlighting how central surveillance data are for our understanding of *Vibrio* dynamics. Furthermore, genes related to transport mechanisms, in-depth analysis into the gene expression of the accessory genes of interest identified here and *in situ* exploration into *Vibrio*–plankton host relationships, will need to be explored to fully understand the dispersal potential during El Niño events. Quantifying such effects of climate phenomena, such as El Niño events, on water-borne diseases will increase our predictive potential to forecast seasonal and inter-annual variations of subsequent infections, amidst predicted increased frequency of El Niño events [54] and wider climate change, to ultimately mitigate the effect on human health.

## supplementary material

10.1099/mgen.0.001317 Uncited Supplementary Material 1.

## Abbreviations

ENSO: El Niño Southern Oscillation
MCMC: Markov chain Monte Carlo
MEI: multivariate ENSO index
NOAA: National Oceanic and Atmospheric Administration
SST: sea surface temperature
T3SS: type III secretion system
Vp: *Vibrio parahaemolyticus*
VpST3: *Vibrio parahaemolyticus* sequence type 3
