# Supplementary material for: Identifying gene-level mechanisms of successful dispersal of Vibrio parahaemolyticus during El Niño events
Source: Microb Genom. 2024 Nov 8;10(11):001317. doi: 10.1099/mgen.0.001317 (PMC11547132; doi:10.1099/mgen.0.001317)
Supplement: Uncited Supplementary Material 1. [file mgen-10-01317-s001.pdf]

Supplementary Information for Identifying gene-level mechanisms of successful dispersal of *Vibrio parahaemolyticus* during El Niño events

Amy Marie Campbell[1,2], Ronnie G. Gavilan[3,4], Chris Hauton[1], Ronny van Aerle[2], and Jaime Martinez-Urtaza[2,4*]

[1] School of Ocean and Earth Science, University of Southampton, National Oceanography Centre, Southampton, UK

[2] Centre for Environment, Fisheries and Aquaculture Science (CEFAS), Weymouth, UK

[3] Centro Nacional de Salud Pública, Instituto Nacional de Salud, Lima, Peru. (rgavilan@ins.gob.pe)

[4] Department of Genetics and Microbiology, Autonomous University of Barcelona, Barcelona, Spain

Supplementary Figures

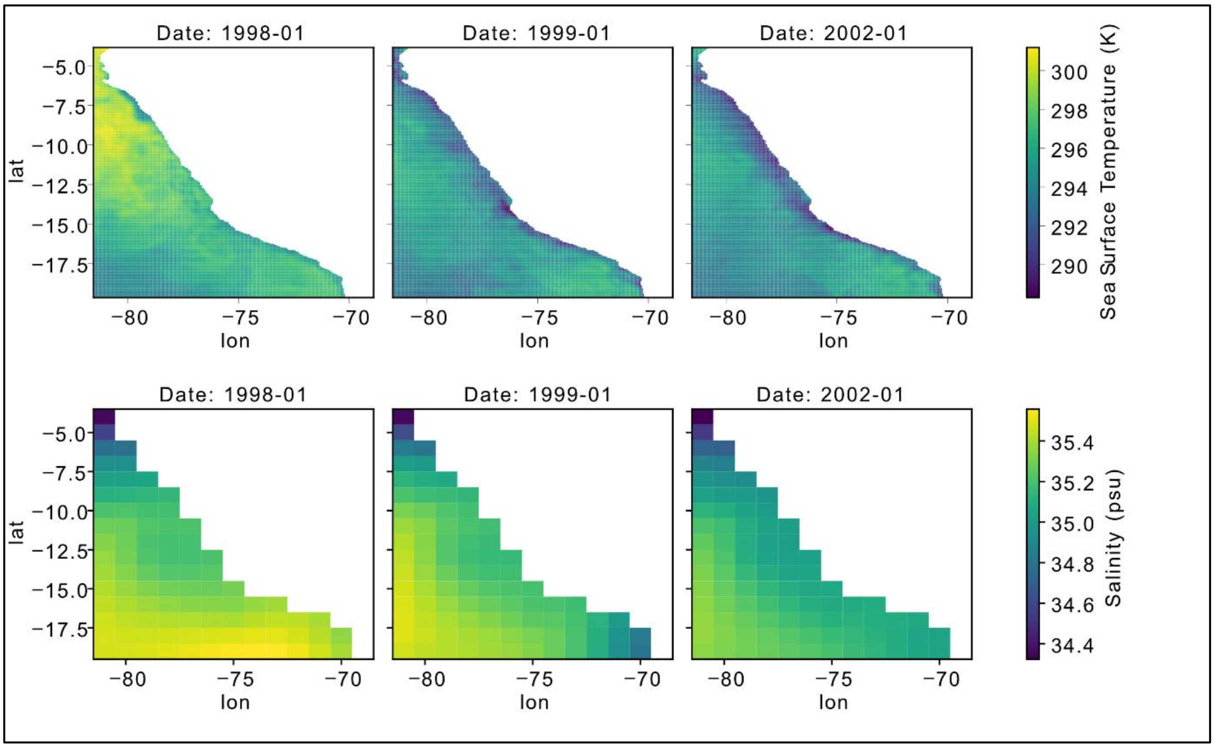

Supplementary Figure S1: Effect of El Niño events on the sea surface temperature (top) and salinity (bottom) of the Peru coastline, comparing the month of January across (left) a strong El Niño event with an MEI of 2.2 in 1998, (middle) a La Niña event with an MEI of -1.3 in 1999, (right) normal conditions with an MEI of 0.1 in 2002.

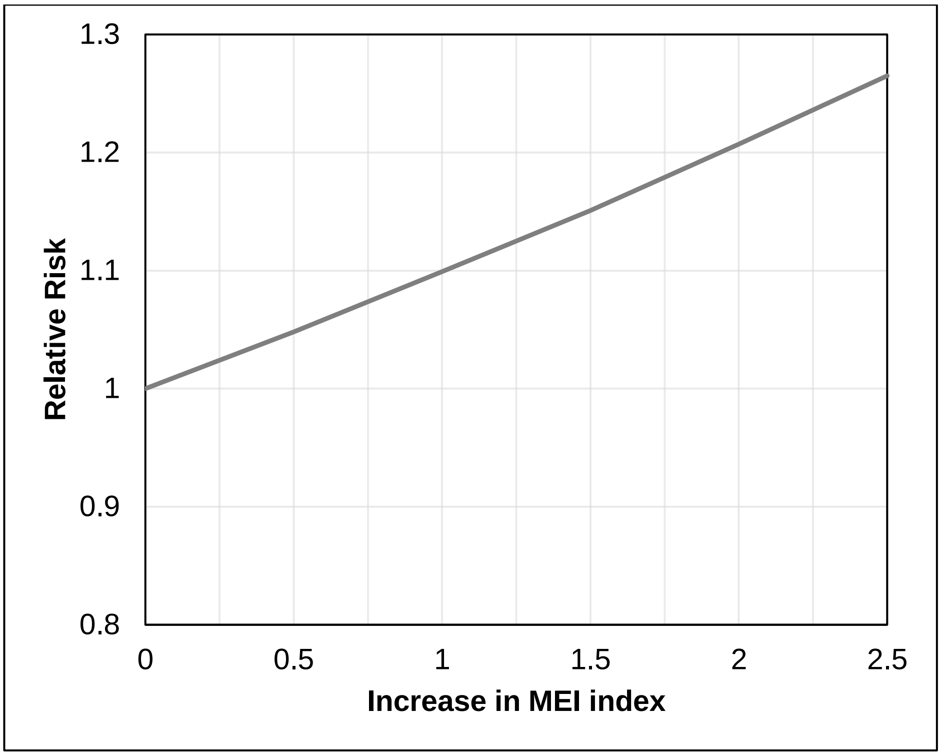

Supplementary Figure S2: Generalised linear model of relative risk (RR) association between MEI index and increases in Vp detection (relative risk) with no lagged effects accounted for

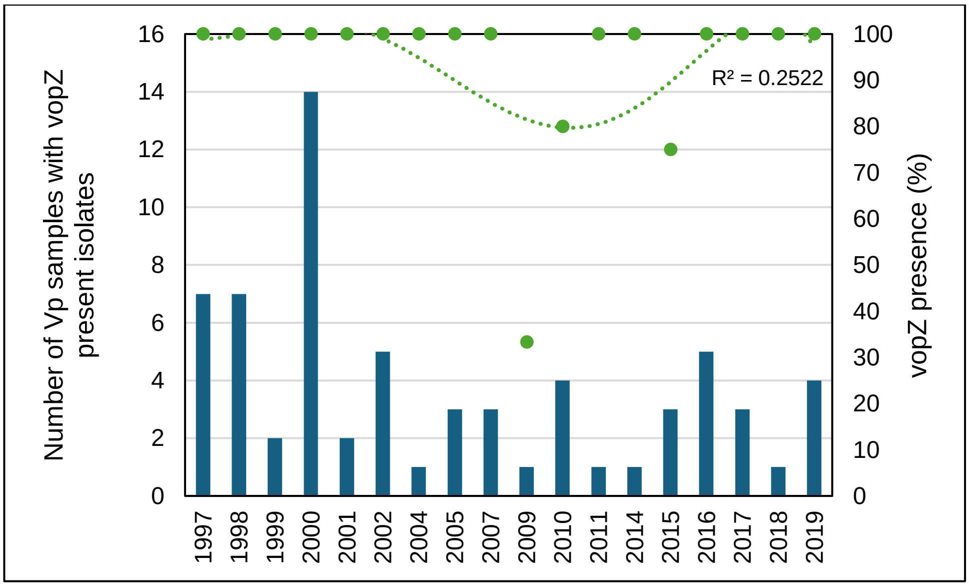

Supplementary Figure S3: Annual number of VpST3 samples isolated in South America with *vopZ* present, and annual percentage presence over time, with a fifth-order polynomial trendline indicating no linear trend.

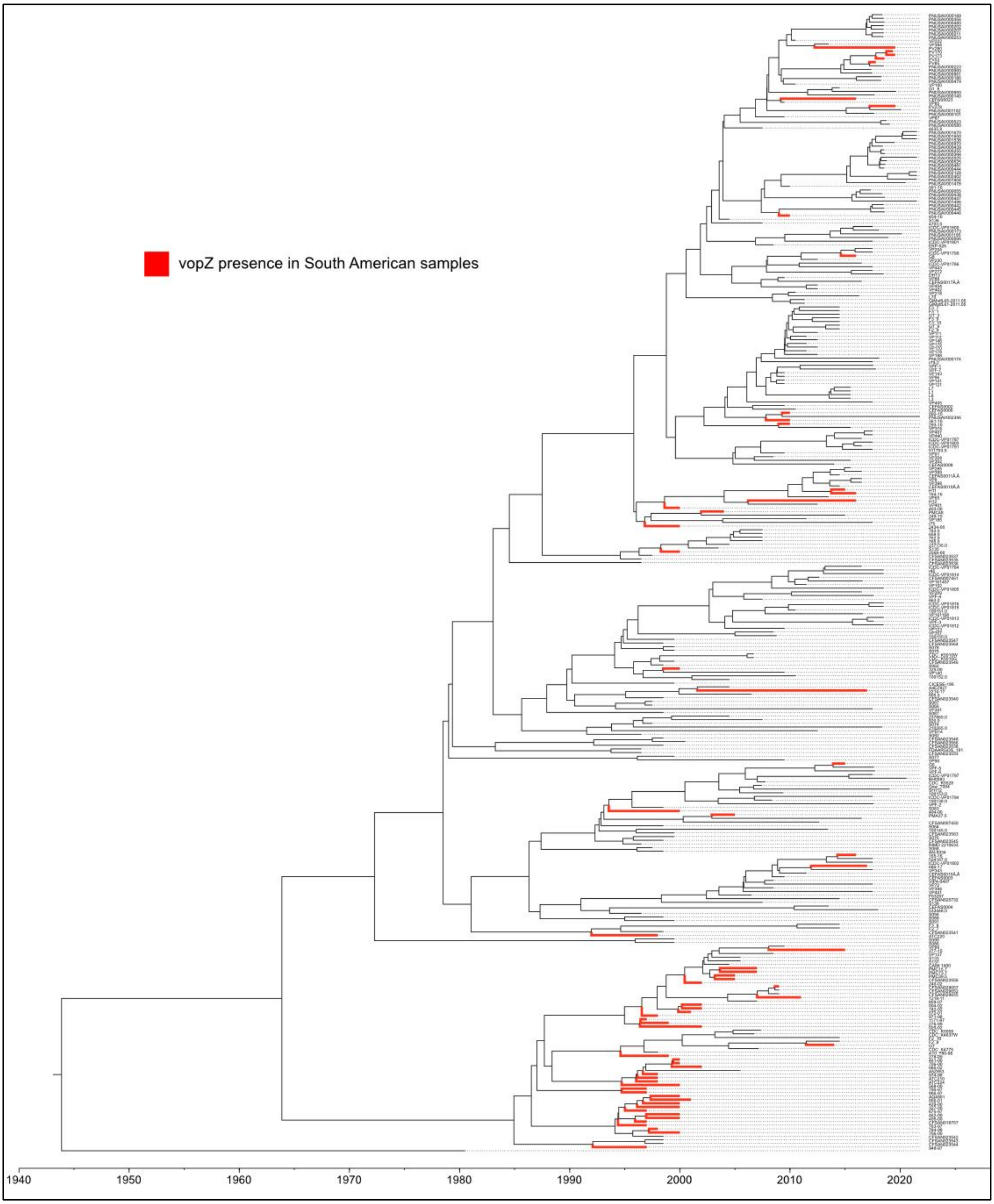

Supplementary Figure S4: *vopZ* presence in South American VpST3 samples situated within a global phylogeny from Campbell et al., (2024) to demonstrate lineage-independence (full methodology for globally phylogeny found in Campbell et al., (2024))

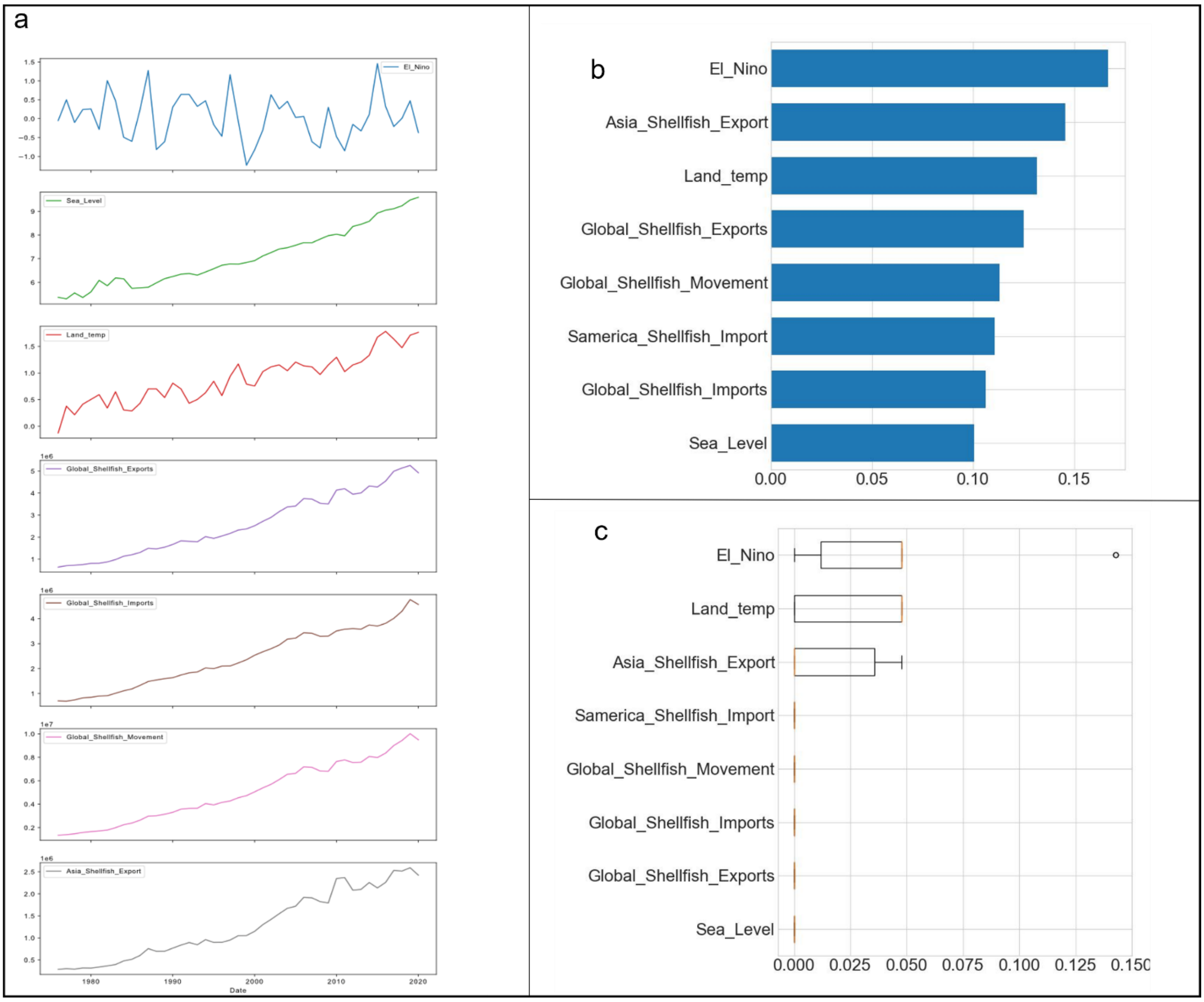

Supplementary Figure S5: Time series of driver data used to train the random forest model (a), feature gini importance (b) and permutation importance (c) of variables used to predict *vopZ* in the random forest classification model.

Supplementary Tables

Supplementary Table S1: Isolates reported to the Instituto Nacional de Salud (Lima, Peru) between 1994-2017 from hospitals and public health laboratories

Origin	Department (City)	Date of Isolation	Year	Type of Sample	Species
Hospital Huacho	Lima	02/01/1994	1994	Faecal	Vibrio parahaemolyticus
Hospital Huacho	Lima	02/01/1994	1994	Faecal	Vibrio parahaemolyticus
Hospital Huacho	Lima	02/01/1994	1994	Faecal	Vibrio parahaemolyticus
Hospital Maria Auxiliadora	Lima	08/03/1995	1995	Faecal	Vibrio parahaemolyticus
Hospital Maria Auxiliadora	Lima	19/02/1995	1995	Faecal	Vibrio parahaemolyticus
Hospital Maria Auxiliadora	Lima	16/02/1995	1995	Faecal	Vibrio parahaemolyticus
Hospital Maria Auxiliadora	Lima	06/03/1995	1995	Faecal	Vibrio parahaemolyticus
Hospital Maria Auxiliadora	Lima	19/02/1995	1995	Faecal	Vibrio parahaemolyticus
Hospital Maria Auxiliadora	Lima	31/01/1995	1995	Faecal	Vibrio parahaemolyticus
Hospital Maria Auxiliadora	Lima	30/01/1995	1995	Faecal	Vibrio parahaemolyticus
Hospital Maria Auxiliadora	Lima	18/02/1995	1995	Faecal	Vibrio parahaemolyticus
Hospital Maria Auxiliadora	Lima	03/03/1995	1995	Faecal	Vibrio parahaemolyticus
Hospital Maria Auxiliadora	Lima	25/01/1995	1995	Faecal	Vibrio parahaemolyticus
Hospital Maria Auxiliadora	Lima	21/02/1995	1995	Faecal	Vibrio parahaemolyticus
Hospital Maria Auxiliadora	Lima	17/04/1995	1995	Faecal	Vibrio parahaemolyticus
Hospital Maria Auxiliadora	Lima	17/04/1995	1995	Faecal	Vibrio parahaemolyticus
Hospital Maria Auxiliadora	Lima	17/04/1995	1995	Faecal	Vibrio parahaemolyticus
Hospital Maria Auxiliadora	Lima	10/04/1995	1995	Faecal	Vibrio parahaemolyticus
Hospital Maria Auxiliadora	Lima	10/04/1995	1995	Faecal	Vibrio parahaemolyticus
Hospital Maria Auxiliadora	Lima	08/04/1995	1995	Faecal	Vibrio parahaemolyticus
Hospital Maria Auxiliadora	Lima	08/04/1995	1995	Faecal	Vibrio parahaemolyticus
Hospital Maria Auxiliadora	Lima	27/03/1995	1995	Faecal	Vibrio parahaemolyticus
Hospital Maria Auxiliadora	Lima	23/03/1995	1995	Faecal	Vibrio parahaemolyticus
Hospital Maria Auxiliadora	Lima	23/03/1995	1995	Faecal	Vibrio parahaemolyticus
Hospital Maria Auxiliadora	Lima	23/03/1995	1995	Faecal	Vibrio parahaemolyticus
Hospital Maria Auxiliadora	Lima	30/03/1995	1995	Faecal	Vibrio parahaemolyticus
Hospital Maria Auxiliadora	Lima	20/03/1995	1995	Faecal	Vibrio parahaemolyticus
Hospital Maria Auxiliadora	Lima	23/03/1995	1995	Faecal	Vibrio parahaemolyticus
Hospital Maria Auxiliadora	Lima	15/03/1995	1995	Faecal	Vibrio parahaemolyticus
Hospital Maria Auxiliadora	Lima	14/03/1995	1995	Faecal	Vibrio parahaemolyticus
Hospital Maria Auxiliadora	Lima	14/03/1995	1995	Faecal	Vibrio parahaemolyticus
Hospital Maria Auxiliadora	Lima	14/03/1995	1995	Faecal	Vibrio parahaemolyticus
Hospital Maria Auxiliadora	Lima	13/02/1995	1995	Faecal	Vibrio parahaemolyticus
Hospital Maria Auxiliadora	Lima	13/03/1995	1995	Faecal	Vibrio parahaemolyticus
Hospital Maria Auxiliadora	Lima	13/03/1995	1995	Faecal	Vibrio parahaemolyticus
Hospital Maria Auxiliadora	Lima	13/03/1995	1995	Faecal	Vibrio parahaemolyticus
Hospital Maria Auxiliadora	Lima	12/03/1995	1995	Faecal	Vibrio parahaemolyticus
Hospital Maria Auxiliadora	Lima	13/03/1995	1995	Faecal	Vibrio parahaemolyticus
Hospital Maria Auxiliadora	Lima	17/03/1995	1995	Faecal	Vibrio parahaemolyticus
Hospital Maria Auxiliadora	Lima	17/03/1995	1995	Faecal	Vibrio parahaemolyticus
Hospital Maria Auxiliadora	Lima	05/04/1995	1995	Faecal	Vibrio parahaemolyticus
Hospital Maria Auxiliadora	Lima	05/04/1995	1995	Faecal	Vibrio parahaemolyticus
Hospital Maria Auxiliadora	Lima	07/04/1995	1995	Faecal	Vibrio parahaemolyticus
Hospital Maria Auxiliadora	Lima	08/04/1995	1995	Faecal	Vibrio parahaemolyticus
Hospital Maria Auxiliadora	Lima	10/04/1995	1995	Faecal	Vibrio parahaemolyticus
Hospital Maria Auxiliadora	Lima	21/04/1995	1995	Faecal	Vibrio parahaemolyticus
H. Las Mercedes Chiclayo	Lambayeque	18/02/1996	1996	Faecal	Vibrio parahaemolyticus
H. La Caleta Chimbote	Ancash	07/03/1996	1996	Faecal	Vibrio parahaemolyticus
H. La Caleta Chimbote	Ancash	12/03/1996	1996	Faecal	Vibrio parahaemolyticus
H. La Caleta Chimbote	Ancash	09/03/1996	1996	Faecal	Vibrio parahaemolyticus
H. La Caleta Chimbote	Ancash	08/03/1996	1996	Faecal	Vibrio parahaemolyticus
H. Las Mercedes Chiclayo	Lambayeque	10/03/1996	1996	Faecal	Vibrio parahaemolyticus
H. Las Mercedes Chiclayo	Lambayeque	22/04/1996	1996	Faecal	Vibrio parahaemolyticus
Hospital Maria Auxiliadora	Lima	29/01/1996	1996	Faecal	Vibrio parahaemolyticus
Hospital Maria Auxiliadora	Lima	22/02/1996	1996	Faecal	Vibrio parahaemolyticus
H. Las Mercedes Chiclayo	Lambayeque	12/09/1996	1996	Faecal	Vibrio parahaemolyticus
H. Las Mercedes	Lambayeque	02/01/1997	1997	Faecal	Vibrio parahaemolyticus
H. Las Mercedes	Lambayeque	22/02/1997	1997	Faecal	Vibrio parahaemolyticus
H. Las Mercedes	Lambayeque	25/02/1997	1997	Faecal	Vibrio parahaemolyticus
H. Chancay	Lima	05/07/1997	1997	Faecal	Vibrio parahaemolyticus
AGO Lima	Lima	01/08/1997	1997	Faecal	Vibrio parahaemolyticus
H. Emergencias Pediatricas	Lima	02/08/1997	1997	Faecal	Vibrio parahaemolyticus
H. Emergencias Pediatricas	Lima	08/08/1997	1997	Faecal	Vibrio parahaemolyticus
H. Emergencias Pediatricas	Lima	11/08/1997	1997	Faecal	Vibrio parahaemolyticus
H. Las Mercedes	Lambayeque	22/07/1997	1997	Faecal	Vibrio parahaemolyticus
LR Cajamarca	Cajamarca	09/08/1997	1997	Faecal	Vibrio parahaemolyticus
LR Cajamarca	Cajamarca	09/08/1997	1997	Faecal	Vibrio parahaemolyticus
LR Cajamarca	Cajamarca	14/08/1997	1997	Faecal	Vibrio parahaemolyticus
LR Cajamarca	Cajamarca	17/08/1997	1997	Faecal	Vibrio parahaemolyticus
H. Emergencias Pediatricas	Lima	02/09/1997	1997	Faecal	Vibrio parahaemolyticus
H. Emergencias Pediatricas	Lima	02/09/1997	1997	Faecal	Vibrio parahaemolyticus
LR Cajamarca	Cajamarca	03/09/1997	1997	Faecal	Vibrio parahaemolyticus
LR Lambayeque	Lambayeque	27/08/1997	1997	Faecal	Vibrio parahaemolyticus
H. Emergencias Pediatricas	Lima	04/05/1997	1997	Faecal	Vibrio parahaemolyticus
H. Carrion	Lima	19/09/1997	1997	Faecal	Vibrio parahaemolyticus
H. Carrion	Lima	15/09/1997	1997	Faecal	Vibrio parahaemolyticus
LR Lambayeque	Lambayeque	26/08/1997	1997	Faecal	Vibrio parahaemolyticus
LR Lambayeque	Lambayeque	07/08/1997	1997	Faecal	Vibrio parahaemolyticus
H. Carrion	Lima	11/09/1997	1997	Faecal	Vibrio parahaemolyticus
H. Carrion	Lima	11/09/1997	1997	Faecal	Vibrio parahaemolyticus
H. Carrion	Lima	11/09/1997	1997	Faecal	Vibrio parahaemolyticus
H. Carrion	Lima	11/09/1997	1997	Faecal	Vibrio parahaemolyticus
H. Carrion	Lima	11/09/1997	1997	Faecal	Vibrio parahaemolyticus
H. Carrion	Lima	11/09/1997	1997	Faecal	Vibrio parahaemolyticus
H. Carrion	Lima	13/09/1997	1997	Faecal	Vibrio parahaemolyticus
H. Carrion	Lima	11/09/1997	1997	Faecal	Vibrio parahaemolyticus
H. Carrion	Lima	11/09/1997	1997	Faecal	Vibrio parahaemolyticus
H. Carrion	Lima	03/09/1997	1997	Faecal	Vibrio parahaemolyticus
H. Carrion	Lima	15/09/1997	1997	Faecal	Vibrio parahaemolyticus
H. Carrion	Lima	08/09/1997	1997	Faecal	Vibrio parahaemolyticus
H. Carrion	Lima	08/09/1997	1997	Faecal	Vibrio parahaemolyticus
H. Carrion	Lima	08/09/1997	1997	Faecal	Vibrio parahaemolyticus
H. Carrion	Lima	03/09/1997	1997	Faecal	Vibrio parahaemolyticus
H. Carrion	Lima	03/09/1997	1997	Faecal	Vibrio parahaemolyticus
H. Carrion	Lima	04/09/1997	1997	Faecal	Vibrio parahaemolyticus
H. Carrion	Lima	08/09/1997	1997	Faecal	Vibrio parahaemolyticus
H. Carrion	Lima	08/09/1997	1997	Faecal	Vibrio parahaemolyticus
LR Cajamarca	Cajamarca	18/09/1997	1997	Faecal	Vibrio parahaemolyticus
LR Cajamarca	Cajamarca	25/09/1997	1997	Faecal	Vibrio parahaemolyticus
H. Puente Piedra	Lima	20/10/1997	1997	Faecal	Vibrio parahaemolyticus
H. Puente Piedra	Lima	20/10/1997	1997	Faecal	Vibrio parahaemolyticus
LR Lambayeque	Lambayeque	12/09/1997	1997	Faecal	Vibrio parahaemolyticus
LR Lima Este	Lima	11/10/1997	1997	Faecal	Vibrio parahaemolyticus
LR Huaras	Ancash	15/10/1997	1997	Faecal	Vibrio parahaemolyticus

LR Cajamarca	Cajamarca	30/10/1997	1997	Faecal	Vibrio parahaemolyticus
H. Maria Auxiliadora	Lima	16/09/1997	1997	Faecal	Vibrio parahaemolyticus
H. Maria Auxiliadora	Lima	18/08/1997	1997	Faecal	Vibrio parahaemolyticus
H. Maria Auxiliadora	Lima	21/10/1997	1997	Faecal	Vibrio parahaemolyticus
H. Maria Auxiliadora	Lima	21/10/1997	1997	Faecal	Vibrio parahaemolyticus
H. Puente Piedra	Lima	17/11/1997	1997	Faecal	Vibrio parahaemolyticus
H. Emergencias Pediatricas	Lima	13/11/1997	1997	Faecal	Vibrio parahaemolyticus
H. Emergencias Pediatricas	Lima	15/11/1997	1997	Faecal	Vibrio parahaemolyticus
LR La Libertad	La_Libertad	18/09/1997	1997	Faecal	Vibrio parahaemolyticus
LR La Libertad	La_Libertad	28/09/1997	1997	Faecal	Vibrio parahaemolyticus
LR Moquegua	Moquegua	12/11/1997	1997	Faecal	Vibrio parahaemolyticus
LR Moquegua	Moquegua	02/11/1997	1997	Faecal	Vibrio parahaemolyticus
H. Huaral	Lima	16/11/1997	1997	Faecal	Vibrio parahaemolyticus
H. Puente Piedra	Lima	10/12/1997	1997	Faecal	Vibrio parahaemolyticus
LR Moquegua	Moquegua	30/11/1997	1997	Faecal	Vibrio parahaemolyticus
LR Moquegua	Moquegua	30/11/1997	1997	Faecal	Vibrio parahaemolyticus
LR Moquegua	Moquegua	27/11/1997	1997	Faecal	Vibrio parahaemolyticus
LR Moquegua	Moquegua	27/11/1997	1997	Faecal	Vibrio parahaemolyticus
LR Lambayeque	Lambayeque	20/10/1997	1997	Faecal	Vibrio parahaemolyticus
LR Lambayeque	Lambayeque	14/11/1997	1997	Faecal	Vibrio parahaemolyticus
LR Lima Este	Lima	16/12/1997	1997	Faecal	Vibrio parahaemolyticus
LR Lima Ciudad	Lima	16/12/1997	1997	Faecal	Vibrio parahaemolyticus
LR Lima Ciudad	Lima	03/12/1997	1997	Faecal	Vibrio parahaemolyticus
H. Puente Piedra	Lima	29/12/1997	1998	Faecal	Vibrio parahaemolyticus
H. Puente Piedra	Lima	29/12/1997	1998	Faecal	Vibrio parahaemolyticus
H. Huaral	Lima	24/12/1997	1998	Faecal	Vibrio parahaemolyticus
H. Maria Auxiliadora	Lima	18/11/1997	1998	Faecal	Vibrio parahaemolyticus
H. Puente Piedra	Lima	08/01/1998	1998	Faecal	Vibrio parahaemolyticus
H. Puente Piedra	Lima	08/01/1998	1998	Faecal	Vibrio parahaemolyticus
H. Puente Piedra	Lima	13/01/1998	1998	Faecal	Vibrio parahaemolyticus
H. Sergio Bernales	Lima	03/12/1998	1998	Faecal	Vibrio parahaemolyticus
H. Sergio Bernales	Lima	03/12/1998	1998	Faecal	Vibrio parahaemolyticus
H. Emergencias Pediatricas	Lima	12/01/1998	1998	Faecal	Vibrio parahaemolyticus
H. Huaraz	Ancash	30/01/1998	1998	Faecal	Vibrio parahaemolyticus
H. Huaraz	Ancash	28/01/1998	1998	Faecal	Vibrio parahaemolyticus
H. Huaraz	Ancash	05/12/1997	1998	Faecal	Vibrio parahaemolyticus
H. Huaraz	Ancash	05/12/1997	1998	Faecal	Vibrio parahaemolyticus
H. Huaraz	Ancash	04/12/1997	1998	Faecal	Vibrio parahaemolyticus
H. Huaraz	Ancash	27/11/1997	1998	Faecal	Vibrio parahaemolyticus
H. Emergencias Pediatricas	Lima	01/02/1998	1998	Faecal	Vibrio parahaemolyticus
LR San Martin	San_Martin	24/01/1998	1998	Faecal	Vibrio parahaemolyticus
H. Puente Piedra	Lima	22/01/1998	1998	Faecal	Vibrio parahaemolyticus
H. Puente Piedra	Lima	04/02/1998	1998	Faecal	Vibrio parahaemolyticus
H. Puente Piedra	Lima	10/02/1998	1998	Faecal	Vibrio parahaemolyticus
H. Puente Piedra	Lima	10/02/1998	1998	Faecal	Vibrio parahaemolyticus
H. Puente Piedra	Lima	10/02/1998	1998	Faecal	Vibrio parahaemolyticus
LR Cajamarca	Cajamarca	13/11/1997	1998	Faecal	Vibrio parahaemolyticus
LR Cajamarca	Cajamarca	09/12/1997	1998	Faecal	Vibrio parahaemolyticus
LR Cajamarca	Cajamarca	16/01/1998	1998	Faecal	Vibrio parahaemolyticus
H. Emergencias Pediatricas	Lima	11/02/1998	1998	Faecal	Vibrio parahaemolyticus
H. Emergencias Pediatricas	Lima	11/02/1998	1998	Faecal	Vibrio parahaemolyticus
H. Puente Piedra	Lima	14/02/1998	1998	Faecal	Vibrio parahaemolyticus
H. Puente Piedra	Lima	18/02/1998	1998	Faecal	Vibrio parahaemolyticus
H. Puente Piedra	Lima	23/02/1998	1998	Faecal	Vibrio parahaemolyticus
H. Puente Piedra	Lima	23/02/1998	1998	Faecal	Vibrio parahaemolyticus
LR Lima Ciudad	Lima	26/02/1998	1998	Faecal	Vibrio parahaemolyticus
LR Lima Ciudad	Lima	23/02/1998	1998	Faecal	Vibrio parahaemolyticus
LR Lima Ciudad	Lima	23/02/1998	1998	Faecal	Vibrio parahaemolyticus
LR Lima Ciudad	Lima	23/02/1998	1998	Faecal	Vibrio parahaemolyticus
LR Lima Ciudad	Lima	23/02/1998	1998	Faecal	Vibrio parahaemolyticus
H. Puente Piedra	Lima	01/03/1998	1998	Faecal	Vibrio parahaemolyticus
LR Tumbes	Tumbes	28/02/1998	1998	Faecal	Vibrio parahaemolyticus
LR Tumbes	Tumbes	28/02/1998	1998	Faecal	Vibrio parahaemolyticus
LR Tumbes	Tumbes	28/02/1998	1998	Faecal	Vibrio parahaemolyticus
H. Dos de Mayo	Lima	27/02/1998	1998	Faecal	Vibrio parahaemolyticus
H. Dos de Mayo	Lima	27/02/1998	1998	Faecal	Vibrio parahaemolyticus
LR Cajamarca	Cajamarca	09/02/1998	1998	Faecal	Vibrio parahaemolyticus
LR Cajamarca	Cajamarca	06/02/1998	1998	Faecal	Vibrio parahaemolyticus
LR Cajamarca	Cajamarca	06/02/1998	1998	Faecal	Vibrio parahaemolyticus
LR Cajamarca	Cajamarca	04/02/1998	1998	Faecal	Vibrio parahaemolyticus
LR Cajamarca	Cajamarca	04/02/1998	1998	Faecal	Vibrio parahaemolyticus
LR Cajamarca	Cajamarca	04/02/1998	1998	Faecal	Vibrio parahaemolyticus
H. Huaral	Lima	08/02/1998	1998	Faecal	Vibrio parahaemolyticus
H. Puente Piedra	Lima	04/03/1998	1998	Faecal	Vibrio parahaemolyticus
H. Puente Piedra	Lima	05/03/1998	1998	Faecal	Vibrio parahaemolyticus
H. Puente Piedra	Lima	09/03/1998	1998	Faecal	Vibrio parahaemolyticus
H. Puente Piedra	Lima	11/03/1998	1998	Faecal	Vibrio parahaemolyticus
H. Trujillo	La_Libertad	16/03/1998	1998	Faecal	Vibrio parahaemolyticus
H. Puente Piedra	Lima	19/03/1998	1998	Faecal	Vibrio parahaemolyticus
H. Sergio Bernales	Lima	05/02/1998	1998	Faecal	Vibrio parahaemolyticus
H. Sergio Bernales	Lima	09/02/1998	1998	Faecal	Vibrio parahaemolyticus
H. Sergio Bernales	Lima	10/02/1998	1998	Faecal	Vibrio parahaemolyticus
H. Sergio Bernales	Lima	11/02/1998	1998	Faecal	Vibrio parahaemolyticus
H. Puente Piedra	Lima	23/03/1998	1998	Faecal	Vibrio parahaemolyticus
H. Emergencias Pediatricas	Lima	23/03/1998	1998	Faecal	Vibrio parahaemolyticus
H. Lambayeque	Lambayeque	18/02/1998	1998	Faecal	Vibrio parahaemolyticus
H. Huancavelica	Huancavelica	01/02/1998	1998	Faecal	Vibrio parahaemolyticus
H. Huacho	Lima	22/01/1998	1998	Faecal	Vibrio parahaemolyticus
H. Huacho	Lima	23/01/1998	1998	Faecal	Vibrio parahaemolyticus
H. Lambayeque	Lambayeque	19/03/1998	1998	Faecal	Vibrio parahaemolyticus
H. Puente Piedra	Lima	30/04/1998	1998	Faecal	Vibrio parahaemolyticus
H. Puente Piedra	Lima	06/05/1998	1998	Faecal	Vibrio parahaemolyticus
H. Hipolito Hunanue	Lima	14/04/1998	1998	Faecal	Vibrio parahaemolyticus
H. Hipolito Hunanue	Lima	14/04/1998	1998	Faecal	Vibrio parahaemolyticus
H. Dos de Mayo	Lima	28/04/1998	1998	Faecal	Vibrio parahaemolyticus
LR Loreto	Loreto	30/04/1998	1998	Faecal	Vibrio parahaemolyticus
H. Arzobispo Loayza	Lima	11/10/1998	1998	Faecal	Vibrio parahaemolyticus
H. Lambayeque	Lambayeque	25/09/1998	1998	Faecal	Vibrio parahaemolyticus
H. Daniel Alcides Carrion	Lima	13/10/1998	1998	Faecal	Vibrio parahaemolyticus
H. Daniel Alcides Carrion	Lima	20/08/1998	1998	Faecal	Vibrio parahaemolyticus
H. Puente Piedra	Lima	03/02/1999	1999	Faecal	Vibrio parahaemolyticus
H. Puente Piedra	Lima	03/02/1999	1999	Faecal	Vibrio parahaemolyticus
H. Puente Piedra	Lima	06/02/1999	1999	Faecal	Vibrio parahaemolyticus
H. Puente Piedra	Lima	09/02/1999	1999	Faecal	Vibrio parahaemolyticus
H. Puente Piedra	Lima	11/02/1999	1999	Faecal	Vibrio parahaemolyticus
LR Loreto	Loreto	09/01/1999	1999	Faecal	Vibrio parahaemolyticus
H. Emergencias Pediatricas	Lima	06/03/1999	1999	Faecal	Vibrio parahaemolyticus
LR Lambayeque	Lambayeque	16/02/1999	1999	Faecal	Vibrio parahaemolyticus
LR Lambayeque	Lambayeque	15/03/1999	1999	Faecal	Vibrio parahaemolyticus
LR Lambayeque	Lambayeque	24/04/1999	1999	Faecal	Vibrio parahaemolyticus
LR Lambayeque	Lambayeque	19/08/1999	1999	Faecal	Vibrio parahaemolyticus
LR Lambayeque	Lambayeque	15/09/1999	1999	Faecal	Vibrio parahaemolyticus
H. Huaral	Lima	18/01/2000	2000	Faecal	Vibrio parahaemolyticus
LR Ancash	Ancash	14/01/2000	2000	Faecal	Vibrio parahaemolyticus
LR Lima Este	Lima	08/03/2000	2000	Faecal	Vibrio parahaemolyticus
LR Lima Este	Lima	08/03/2000	2000	Faecal	Vibrio parahaemolyticus

LR Lima Este	Lima	08/03/2000	2000	Faecal	Vibrio parahaemolyticus
H. Huaral	Lima	01/03/2000	2000	Faecal	Vibrio parahaemolyticus
LR Lambayeque	Lambayeque	17/02/2000	2000	Faecal	Vibrio parahaemolyticus
LR Lambayeque	Lambayeque	17/02/2000	2000	Faecal	Vibrio parahaemolyticus
LR Lambayeque	Lambayeque	17/02/2000	2000	Faecal	Vibrio parahaemolyticus
LR Lima Este	Lima	13/04/2000	2000	Faecal	Vibrio parahaemolyticus
LR Lima Este	Lima	13/04/2000	2000	Faecal	Vibrio parahaemolyticus
H. Collique	Lima	23/03/2000	2000	Faecal	Vibrio parahaemolyticus
H. Collique	Lima	23/03/2000	2000	Faecal	Vibrio parahaemolyticus
H. Dos de Mayo	Lima	24/03/2000	2000	Faecal	Vibrio parahaemolyticus
LR Loreto	Loreto	17/04/2000	2000	Faecal	Vibrio parahaemolyticus
LR Lima Este	Lima	05/06/2000	2000	Faecal	Vibrio parahaemolyticus
LR Lima Este	Lima	05/06/2000	2000	Faecal	Vibrio parahaemolyticus
LR Lambayeque	Lambayeque	09/06/2000	2000	Faecal	Vibrio parahaemolyticus
H. Huaral	Lima	05/06/2000	2000	Faecal	Vibrio parahaemolyticus
LR Loreto	Loreto	14/06/2000	2000	Faecal	Vibrio parahaemolyticus
LR Lima Este	Lima	19/10/2000	2000	Faecal	Vibrio parahaemolyticus
LR Lambayeque	Lambayeque	30/10/2000	2000	Faecal	Vibrio parahaemolyticus
LR Lima Este	Lima	07/11/2000	2000	Faecal	Vibrio parahaemolyticus
LR Loreto	Loreto	19/10/2000	2000	Faecal	Vibrio parahaemolyticus
LR Cajamarca	Cajamarca	26/09/2000	2000	Faecal	Vibrio parahaemolyticus
LR Lima Este	Lima	19/12/2000	2000	Faecal	Vibrio parahaemolyticus
Collique Sergio Bernales	Lima	07/02/2001	2001	Faecal	Vibrio parahaemolyticus
LR Lambayeque	Lambayeque	02/01/2001	2001	Faecal	Vibrio parahaemolyticus
LR Lima-Sur	Lima	05/03/2001	2001	Faecal	Vibrio parahaemolyticus
LR Lima-Sur	Lima	08/03/2001	2001	Faecal	Vibrio parahaemolyticus
H. Huaral	Lima	27/03/2001	2001	Faecal	Vibrio parahaemolyticus
H. Chiclayo	Lambayeque	28/05/2001	2001	Faecal	Vibrio parahaemolyticus
H. Chiclayo	Lambayeque	28/05/2001	2001	Faecal	Vibrio parahaemolyticus
LR Lima Este	Lima	29/05/2001	2001	Faecal	Vibrio parahaemolyticus
LR Lima Este	Lima	29/05/2001	2001	Faecal	Vibrio parahaemolyticus
LR Lima Este	Lima	29/05/2001	2001	Faecal	Vibrio parahaemolyticus
LR Lima Este	Lima	29/05/2001	2001	Faecal	Vibrio parahaemolyticus
LR Barranco	Lima	20/06/2001	2001	Faecal	Vibrio parahaemolyticus
LR Barranco	Lima	20/06/2001	2001	Faecal	Vibrio parahaemolyticus
LR Iquitos	Loreto	10/07/2001	2001	Faecal	Vibrio parahaemolyticus
LR Iquitos	Loreto	10/07/2001	2001	Faecal	Vibrio parahaemolyticus
LR Iquitos	Loreto	18/07/2001	2001	Faecal	Vibrio parahaemolyticus
LR Iquitos	Loreto	18/07/2001	2001	Faecal	Vibrio parahaemolyticus
LR Iquitos	Loreto	11/07/2001	2001	Faecal	Vibrio parahaemolyticus
LR Iquitos	Loreto	11/07/2001	2001	Faecal	Vibrio parahaemolyticus
LR Iquitos	Loreto	11/07/2001	2001	Faecal	Vibrio parahaemolyticus
LR Arequipa	Arequipa	18/07/2001	2001	Faecal	Vibrio parahaemolyticus
LR Arequipa	Arequipa	18/07/2001	2001	Faecal	Vibrio parahaemolyticus
LR Loreto	Loreto	27/09/2001	2001	Faecal	Vibrio parahaemolyticus
LR Lima-Sur	Lima	06/11/2001	2001	Faecal	Vibrio parahaemolyticus
LR Lima Este	Lima	20/02/2002	2002	Faecal	Vibrio parahaemolyticus
H. Dos de Mayo	Lima	20/02/2002	2002	Faecal	Vibrio parahaemolyticus
H. Chiclayo	Lambayeque	22/02/2002	2002	Faecal	Vibrio parahaemolyticus
H. Chiclayo	Lambayeque	15/05/2002	2002	Faecal	Vibrio parahaemolyticus
H. Sergio Bernales	Lima	22/03/2002	2002	Faecal	Vibrio parahaemolyticus
H. Sergio Bernales	Lima	26/03/2002	2002	Faecal	Vibrio parahaemolyticus
H. Sergio Bernales	Lima	03/04/2002	2002	Faecal	Vibrio parahaemolyticus
LR Arequipa	Arequipa	16/04/2002	2002	Faecal	Vibrio parahaemolyticus
H. Chiclayo	Lambayeque	16/04/2002	2002	Faecal	Vibrio parahaemolyticus
LR Lima Este	Lima	24/04/2002	2002	Faecal	Vibrio parahaemolyticus
LR Lima Este	Lima	24/04/2002	2002	Faecal	Vibrio parahaemolyticus
LR Iquitos	Loreto	13/05/2002	2002	Faecal	Vibrio parahaemolyticus
H. Chiclayo	Lambayeque	23/05/2002	2002	Faecal	Vibrio parahaemolyticus
H. Chiclayo	Lambayeque	23/05/2002	2002	Faecal	Vibrio parahaemolyticus
LR Lima Este	Lima	12/08/2002	2002	Faecal	Vibrio parahaemolyticus
H. Chiclayo	Lambayeque	24/06/2002	2002	Faecal	Vibrio parahaemolyticus
H. Chiclayo	Lambayeque	24/06/2002	2002	Faecal	Vibrio parahaemolyticus
LR Lima Este	Lima	01/07/2002	2002	Faecal	Vibrio parahaemolyticus
LR Lima Este	Lima	01/07/2002	2002	Faecal	Vibrio parahaemolyticus
LR Iquitos	Loreto	01/07/2002	2002	Faecal	Vibrio parahaemolyticus
LR Lima Ciudad	Lima	27/12/2002	2002	Faecal	Vibrio parahaemolyticus
LR Lima Ciudad	Lima	27/12/2002	2002	Faecal	Vibrio parahaemolyticus
LR Lima Ciudad	Lima	27/12/2002	2002	Faecal	Vibrio parahaemolyticus
LR Lima Ciudad	Lima	27/12/2002	2002	Faecal	Vibrio parahaemolyticus
H. Chiclayo	Lambayeque	14/01/2003	2003	Faecal	Vibrio parahaemolyticus
H. Chiclayo	Lambayeque	14/01/2003	2003	Faecal	Vibrio parahaemolyticus
LR Cajamarca	Cajamarca	31/01/2003	2003	Faecal	Vibrio parahaemolyticus
LR Cajamarca	Cajamarca	31/01/2003	2003	Faecal	Vibrio parahaemolyticus
LR Lima Ciudad	Lima	05/03/2003	2003	Faecal	Vibrio parahaemolyticus
LR Lima Ciudad	Lima	05/03/2003	2003	Faecal	Vibrio parahaemolyticus
LR Lima Ciudad	Lima	05/03/2003	2003	Faecal	Vibrio parahaemolyticus
LR Lima Ciudad	Lima	05/03/2003	2003	Faecal	Vibrio parahaemolyticus
H. Huaral - San Juan Bautista	Lima	14/03/2003	2003	Faecal	Vibrio parahaemolyticus
LR Lima Este	Lima	14/03/2003	2003	Faecal	Vibrio parahaemolyticus
LR Arequipa	Arequipa	23/04/2003	2003	Faecal	Vibrio parahaemolyticus
LR Chimbote	Ancash	28/04/2003	2003	Faecal	Vibrio parahaemolyticus
LR Cajamarca	Cajamarca	16/06/2003	2003	Faecal	Vibrio parahaemolyticus
H. San Bartolome	Lima	18/03/2004	2004	Faecal	Vibrio parahaemolyticus
H. Cayetano Heredia	Lima	17/04/2004	2004	Faecal	Vibrio parahaemolyticus
Lima Sur	Lima	18/01/2005	2005	Faecal	Vibrio parahaemolyticus
Dos de Mayo	Lima	29/01/2005	2005	Faecal	Vibrio parahaemolyticus
Dos de Mayo	Lima	02/01/2005	2005	Faecal	Vibrio parahaemolyticus
Ancash	Ancash	02/04/2005	2005	Faecal	Vibrio parahaemolyticus
Dos deMayo	Lima	15/02/2005	2005	Faecal	Vibrio parahaemolyticus
Huaral	Lima	27/02/2005	2005	Faecal	Vibrio parahaemolyticus
Lambayeque	Lambayeque	09/06/2005	2005	Faecal	Vibrio parahaemolyticus
Huaraz	Ancash	02/10/2006	2006	Faecal	Vibrio parahaemolyticus
Dos de mayo	Lima	21/04/2006	2006	Faecal	Vibrio parahaemolyticus
lima Ciudad	Lima	02/01/2007	2007	Faecal	Vibrio parahaemolyticus
lima Ciudad	Lima	14/02/2007	2007	Faecal	Vibrio parahaemolyticus
Chiclayo	Lambayeque	26/01/2007	2007	Faecal	Vibrio parahaemolyticus
Chiclayo	Lambayeque	02/08/2007	2007	Faecal	Vibrio parahaemolyticus
Chiclayo	Lambayeque	02/08/2007	2007	Faecal	Vibrio parahaemolyticus
Chiclayo	Lambayeque	02/06/2007	2007	Faecal	Vibrio parahaemolyticus
Chiclayo	Lambayeque	02/06/2007	2007	Faecal	Vibrio parahaemolyticus
Dos de Mayo	Lima	03/09/2007	2007	Faecal	Vibrio parahaemolyticus
Dos de Mayo	Lima	03/12/2007	2007	Faecal	Vibrio parahaemolyticus
LARESA LAMBAYEQUE	Lambayeque	19/09/2008	2008	Faecal	Vibrio parahaemolyticus
LAMABAYEQUE	Lambayeque	12/03/2009	2009	Faecal	Vibrio parahaemolyticus
LAMABAYEQUE	Lambayeque	13/03/2009	2009	Faecal	Vibrio parahaemolyticus
LAMABAYEQUE	Lambayeque	14/03/2009	2009	Faecal	Vibrio parahaemolyticus
LAMABAYEQUE	Lambayeque	15/03/2009	2009	Faecal	Vibrio parahaemolyticus
LAMABAYEQUE	Lambayeque	16/03/2009	2009	Faecal	Vibrio parahaemolyticus
LAMABAYEQUE	Lambayeque	17/03/2009	2009	Faecal	Vibrio parahaemolyticus
LAMABAYEQUE	Lambayeque	18/03/2009	2009	Faecal	Vibrio parahaemolyticus
LAMABAYEQUE	Lambayeque	19/03/2009	2009	Faecal	Vibrio parahaemolyticus
LRR Cajamarca	Cajamarca	03/03/2009	2009	Faecal	Vibrio parahaemolyticus
LRR Cajamarca	Cajamarca	03/03/2009	2009	Faecal	Vibrio parahaemolyticus
LRR Cajamarca	Cajamarca	10/03/2009	2009	Faecal	Vibrio parahaemolyticus
LRR Cajamarca	Cajamarca	10/03/2009	2009	Faecal	Vibrio parahaemolyticus

LAMABAYEQUE	Lambayeque	13/03/2009	2009	Faecal	Vibrio parahaemolyticus
LAMABAYEQUE	Lambayeque	14/03/2009	2009	Faecal	Vibrio parahaemolyticus
LAMABAYEQUE	Lambayeque	15/03/2009	2009	Faecal	Vibrio parahaemolyticus
LAMABAYEQUE	Lambayeque	16/03/2009	2009	Faecal	Vibrio parahaemolyticus
Clinica San Borja	Lima	25/02/2009	2009	Faecal	Vibrio parahaemolyticus
Clinica San Borja	Lima	03/03/2009	2009	Faecal	Vibrio parahaemolyticus
LRR Cajamarca	Cajamarca	12/03/2009	2009	Faecal	Vibrio parahaemolyticus
LRR Cajamarca	Cajamarca	12/03/2009	2009	Faecal	Vibrio parahaemolyticus
LRR Cajamarca	Cajamarca	12/03/2009	2009	Faecal	Vibrio parahaemolyticus
LARESA Piura	Piura	16/03/2009	2009	Faecal	Vibrio parahaemolyticus
LAMABAYEQUE	Lambayeque	06/04/2009	2009	Faecal	Vibrio parahaemolyticus
LAMABAYEQUE	Lambayeque	07/04/2009	2009	Faecal	Vibrio parahaemolyticus
LAMABAYEQUE	Lambayeque	08/04/2009	2009	Faecal	Vibrio parahaemolyticus
LAMABAYEQUE	Lambayeque	09/04/2009	2009	Faecal	Vibrio parahaemolyticus
LAMABAYEQUE	Lambayeque	10/04/2009	2009	Faecal	Vibrio parahaemolyticus
LAMABAYEQUE	Lambayeque	11/04/2009	2009	Faecal	Vibrio parahaemolyticus
LAMABAYEQUE	Lambayeque	12/04/2009	2009	Faecal	Vibrio parahaemolyticus
LARESA Piura	Piura	18/03/2009	2009	Faecal	Vibrio parahaemolyticus
HOSPITAL SAN BARTOLOME	Lima	26/03/2009	2009	Faecal	Vibrio parahaemolyticus
Clinica San Borja	Lima	29/04/2009	2009	Faecal	Vibrio parahaemolyticus
LRR CHICLAYO	Lambayeque	11/05/2009	2009	Faecal	Vibrio parahaemolyticus
LRR CHICLAYO	Lambayeque	11/05/2009	2009	Faecal	Vibrio parahaemolyticus
Hospital Emergencia Pediatricas	Lima	03/05/2009	2009	Faecal	Vibrio parahaemolyticus
Hospital Emergencia Pediatricas	Lima	03/05/2009	2009	Faecal	Vibrio parahaemolyticus
LRR CHICLAYO	Lambayeque	18/08/2009	2009	Faecal	Vibrio parahaemolyticus
LRR CHICLAYO	Lambayeque	18/08/2009	2009	Faecal	Vibrio parahaemolyticus
LRR CHICLAYO	Lambayeque	18/08/2009	2009	Faecal	Vibrio parahaemolyticus
HOSPITAL DOS DE MAYO	Lima	28/01/2010	2010	Faecal	Vibrio parahaemolyticus
INS	Lima	25/01/2010	2010	Faecal	Vibrio parahaemolyticus
Hospital Emergencia Pediatricas	Lima	19/01/2010	2010	Faecal	Vibrio parahaemolyticus
CLINICA SAN BORJA	Lima	12/01/2010	2010	Faecal	Vibrio parahaemolyticus
CLINICA SAN BORJA	Lima	20/02/2010	2010	Faecal	Vibrio parahaemolyticus
CLINICA SAN BORJA	Lima	20/02/2010	2010	Faecal	Vibrio parahaemolyticus
CLINICA SAN BORJA	Lima	20/02/2010	2010	Faecal	Vibrio parahaemolyticus
UNIVERSIDAD CAYETANO HEREDIA	Lima	17/02/2010	2010	Faecal	Vibrio parahaemolyticus
Clinica San Borja	Lima	02/03/2010	2010	Faecal	Vibrio parahaemolyticus
LIMA	Lima	07/03/2010	2010	Faecal	Vibrio parahaemolyticus
CLINICA SAN BORJA	Lima	12/03/2010	2010	Faecal	Vibrio parahaemolyticus
INSTITUTO DE SALUD DEL NIÑO	Lima	10/02/2010	2010	Faecal	Vibrio parahaemolyticus
UNIVERSIDAD CAYETANO HEREDIA	Lima	22/04/2010	2010	Faecal	Vibrio parahaemolyticus
CLINICA SAN BORJA	Lima	12/03/2010	2010	Faecal	Vibrio parahaemolyticus
CLINICA SAN BORJA	Lima	28/01/2011	2011	Faecal	Vibrio parahaemolyticus
HOSPITAL SAN BARTOLOME	Lima	23/03/2011	2011	Faecal	Vibrio parahaemolyticus
HOSPITAL SAN BARTOLOME	Lima	06/04/2011	2011	Faecal	Vibrio parahaemolyticus
HOSPITAL DOS DE MAYO	Lima	15/04/2011	2011	Faecal	Vibrio parahaemolyticus
CLINICA SAN BORJA	Lima	03/06/2011	2011	Faecal	Vibrio parahaemolyticus
CLINICA SAN BORJA	Lima	15/03/2012	2012	Faecal	Vibrio parahaemolyticus
CLINICA SAN BORJA	Lima	23/03/2012	2012	Faecal	Vibrio parahaemolyticus
CLINICA SAN BORJA	Lima	02/01/2013	2013	Faecal	Vibrio parahaemolyticus
CLINICA SAN BORJA	Lima	22/01/2013	2013	Faecal	Vibrio parahaemolyticus
CLINICA SAN BORJA	Lima	22/01/2013	2013	Faecal	Vibrio parahaemolyticus
HOSPITAL DOS DE MAYO	Lima	30/12/2013	2014	Faecal	Vibrio parahaemolyticus
CLINICA SAN BORJA	Lima	05/02/2014	2014	Faecal	Vibrio parahaemolyticus
DIRESA PIURA	Piura	09/06/2014	2014	Faecal	Vibrio parahaemolyticus
DIRESA PIURA	Piura	29/10/2014	2014	Faecal	Vibrio parahaemolyticus
CLINICA SAN BORJA	Lima	25/01/2015	2015	Faecal	Vibrio parahaemolyticus
CLINICA SAN BORJA	Lima	16/02/2015	2015	Faecal	Vibrio parahaemolyticus
CLINICA SAN BORJA	Lima	28/03/2015	2015	Faecal	Vibrio parahaemolyticus
CLINICA EL GOLF	Lima	05/04/2015	2015	Faecal	Vibrio parahaemolyticus
CLINICA EL GOLF	Lima	28/03/2015	2015	Faecal	Vibrio parahaemolyticus
CLINICA SAN BORJA	Lima	23/05/2015	2015	Faecal	Vibrio parahaemolyticus
HOSPITAL SAN BARTOLOME	Lima	24/04/2015	2015	Faecal	Vibrio parahaemolyticus
CLINICA SAN BORJA	Lima	15/06/2015	2015	Faecal	Vibrio parahaemolyticus
HOSPITAL SAN BARTOLOME	Lima	20/06/2015	2015	Faecal	Vibrio parahaemolyticus
CLINICA SAN BORJA	Lima	29/01/2016	2016	Faecal	Vibrio parahaemolyticus
LRR CUSCO	Cusco	15/02/2016	2016	Faecal	Vibrio parahaemolyticus
CLINICA SAN BORJA	Lima	10/02/2016	2016	Faecal	Vibrio parahaemolyticus
CLINICA SAN BORJA	Lima	09/02/2016	2016	Faecal	Vibrio parahaemolyticus
CLINICA SAN BORJA	Lima	06/02/2016	2016	Faecal	Vibrio parahaemolyticus
CLINICA SAN BORJA	Lima	05/02/2016	2016	Faecal	Vibrio parahaemolyticus
CLINICA SAN BORJA	Lima	04/02/2016	2016	Faecal	Vibrio parahaemolyticus
CLINICA SAN BORJA	Lima	07/03/2016	2016	Faecal	Vibrio parahaemolyticus
CLINICA SAN BORJA	Lima	01/04/2016	2016	Faecal	Vibrio parahaemolyticus
CLINICA SAN BORJA	Lima	07/03/2016	2016	Faecal	Vibrio parahaemolyticus
HOSPITAL SAN BARTOLOME	Lima	07/05/2016	2016	Faecal	Vibrio parahaemolyticus
TUMBES	Tumbes	15/02/2017	2017	Faecal	Vibrio parahaemolyticus
CLINICA SAN BORJA	Lima	16/03/2017	2017	Faecal	Vibrio parahaemolyticus
DIRESA PIURA	Piura	12/04/2017	2017	Faecal	Vibrio parahaemolyticus
DIRESA LAMBAYEQUE	Lambayeque	18/04/2017	2017	Faecal	Vibrio parahaemolyticus
CLINICA SAN BORJA	Lima	05/08/2017	2017	Faecal	Vibrio parahaemolyticus
CLINICA SAN BORJA	Lima	17/08/2017	2017	Faecal	Vibrio parahaemolyticus
INSTITUTO DE SALUD	Lima	01/11/2009	2009	Faecal	Vibrio parahaemolyticus
INSTITUTO DE SALUD	Lima	01/11/2009	2009	Faecal	Vibrio parahaemolyticus
INSTITUTO DE SALUD	Piura	01/11/2009	2009	Faecal	Vibrio parahaemolyticus
INSTITUTO DE SALUD	Piura	01/11/2009	2009	Faecal	Vibrio parahaemolyticus
INSTITUTO DE SALUD	Piura	01/11/2009	2009	Faecal	Vibrio parahaemolyticus
INSTITUTO DE SALUD	Piura	01/09/2014	2014	Faecal	Vibrio parahaemolyticus
IMARPE	Lima	03/07/2015	2015	Faecal	Vibrio parahaemolyticus
DIRESA CALLAO	Lima	14/11/2016	2016	Faecal	Vibrio parahaemolyticus
DIRESA CALLAO	Lima	20/02/2017	2017	Faecal	Vibrio parahaemolyticus
NAMRU-6_Arequipa	Arequipa	06/08/1997	1997	Faecal	Vibrio parahaemolyticus
NAMRU-6_Arequipa	Arequipa	05/08/1997	1997	Faecal	Vibrio parahaemolyticus
NAMRU-6_Arequipa	Arequipa	03/09/1997	1997	Faecal	Vibrio parahaemolyticus
NAMRU-6_Arequipa	Arequipa	12/10/1997	1997	Faecal	Vibrio parahaemolyticus
NAMRU-6_Lima-Independencia	Lima	12/01/1998	1998	Faecal	Vibrio parahaemolyticus
NAMRU-6_Lima-Independencia	Lima	01/02/1998	1998	Faecal	Vibrio parahaemolyticus
NAMRU-6_Lima-Independencia	Lima	19/01/1998	1998	Faecal	Vibrio parahaemolyticus
NAMRU-6_Lima-LaVictoria	Lima	01/04/2003	2003	Faecal	Vibrio parahaemolyticus
NAMRU-6_Ancon	Lima	26/01/1998	1998	Faecal	Vibrio parahaemolyticus
NAMRU-6_Ancon	Lima	26/01/1998	1998	Faecal	Vibrio parahaemolyticus
NAMRU-6_Ancon	Lima	27/01/1998	1998	Faecal	Vibrio parahaemolyticus
NAMRU-6_Ancon	Lima	27/01/1998	1998	Faecal	Vibrio parahaemolyticus
NAMRU-6_Ancon	Lima	27/01/1998	1998	Faecal	Vibrio parahaemolyticus
NAMRU-6_Ancon	Lima	26/01/1998	1998	Faecal	Vibrio parahaemolyticus
NAMRU-6_Ancon	Lima	26/01/1998	1998	Faecal	Vibrio parahaemolyticus
NAMRU-6_Ancon	Lima	27/01/1998	1998	Faecal	Vibrio parahaemolyticus
NAMRU-6_Ancon	Lima	27/01/1998	1998	Faecal	Vibrio parahaemolyticus
NAMRU-6_Ancon	Lima	24/01/1998	1998	Faecal	Vibrio parahaemolyticus
NAMRU-6_PampasdeSanJuan	Lima	30/04/1998	1998	Faecal	Vibrio parahaemolyticus
NAMRU-6_PampasdeSanJuan	Lima	30/04/1998	1998	Faecal	Vibrio parahaemolyticus
NAMRU-6_PampasdeSanJuan	Lima	15/04/1998	1998	Faecal	Vibrio parahaemolyticus
NAMRU-6_PampasdeSanJuan	Lima	15/04/1998	1998	Faecal	Vibrio parahaemolyticus
NAMRU-6_-	Lima	01/05/1998	1994	Faecal	Vibrio parahaemolyticus
NAMRU-6_PampasdeSanJuan	Lima	15/04/1998	1998	Faecal	Vibrio parahaemolyticus
NAMRU-6_PampasdeSanJuan	Lima	15/04/1998	1998	Faecal	Vibrio parahaemolyticus
NAMRU-6_PampasdeSanJuan	Lima	15/04/1998	1998	Faecal	Vibrio parahaemolyticus

NAMRU-6_PampasdeSanJuan	Lima	02/05/1998	1998	Faecal	Vibrio parahaemolyticus
NAMRU-6_Lima	Lima	10/02/2001	2001	Faecal	Vibrio parahaemolyticus
NAMRU-6_Lima	Lima	14/03/2001	2001	Faecal	Vibrio parahaemolyticus
NAMRU-6_Lima	Lima	21/02/2001	2001	Faecal	Vibrio parahaemolyticus
NAMRU-6_Lima	Lima	17/12/2013	2013	Faecal	Vibrio parahaemolyticus
NAMRU-6_Lima	Lima	06/03/2017	2017	Faecal	Vibrio parahaemolyticus
NAMRU-6_Lima	Lima	06/01/2014	2014	Faecal	Vibrio parahaemolyticus
NAMRU-6_Lima	Lima	23/01/2014	2014	Faecal	Vibrio parahaemolyticus
NAMRU-6_-	Lima	25/02/2000	2000	Faecal	Vibrio parahaemolyticus
NAMRU-6_-	Lima	11/11/2001	2001	Faecal	Vibrio parahaemolyticus
NAMRU-6_Lima-ISN	Lima	11/02/1994	1994	Faecal	Vibrio parahaemolyticus
NAMRU-6_-	Lima	06/01/1998	1998	Faecal	Vibrio parahaemolyticus
NAMRU-6_-	Lima	03/04/1998	1998	Faecal	Vibrio parahaemolyticus
NAMRU-6_-	Lima	03/04/1998	1998	Faecal	Vibrio parahaemolyticus
NAMRU-6_Lima-HospFAP	Lima	24/01/1998	1998	Faecal	Vibrio parahaemolyticus
NAMRU-6_Lima-HospFAP	Lima	27/01/1998	1998	Faecal	Vibrio parahaemolyticus
NAMRU-6_Lima-HospFAP	Lima	27/01/1998	1998	Faecal	Vibrio parahaemolyticus
NAMRU-6_Lima-HospFAP	Lima	09/02/1998	1998	Faecal	Vibrio parahaemolyticus
NAMRU-6_Lima-HospFAP	Lima	13/02/1998	1998	Faecal	Vibrio parahaemolyticus
NAMRU-6_Lima-HospFAP	Lima	16/02/1998	1998	Faecal	Vibrio parahaemolyticus
NAMRU-6_Lima-HospFAP	Lima	23/02/1998	1998	Faecal	Vibrio parahaemolyticus
NAMRU-6_Lima-HospFAP	Lima	11/02/1998	1998	Faecal	Vibrio parahaemolyticus
NAMRU-6_-	Lima	12/01/1998	1998	Faecal	Vibrio parahaemolyticus
NAMRU-6_-	Lima	05/09/1997	1997	Faecal	Vibrio parahaemolyticus
NAMRU-6_Callao-DAC	Lima	06/04/1998	1998	Faecal	Vibrio parahaemolyticus
NAMRU-6_Lima-HospRebagliati	Lima	16/02/1998	1998	Faecal	Vibrio parahaemolyticus
NAMRU-6_Callao-DAC	Lima	06/04/1998	1998	Faecal	Vibrio parahaemolyticus
NAMRU-6_Callao-DAC	Lima	06/01/1998	1998	Faecal	Vibrio parahaemolyticus
NAMRU-6_Lima-ISN	Lima	08/04/1998	1998	Faecal	Vibrio parahaemolyticus
NAMRU-6_Lima-ISN	Lima	30/04/1998	1998	Faecal	Vibrio parahaemolyticus
NAMRU-6_-	Lima	28/08/1997	1997	Faecal	Vibrio parahaemolyticus
NAMRU-6_Lima-HospMaternoInfantil	Lima	23/03/1998	1998	Faecal	Vibrio parahaemolyticus
NAMRU-6_Lima-HospMaternoInfantil	Lima	16/04/1998	1998	Faecal	Vibrio parahaemolyticus
NAMRU-6_Callao-DAC	Lima	26/04/1998	1998	Faecal	Vibrio parahaemolyticus
NAMRU-6_Callao-DAC	Lima	26/04/1998	1998	Faecal	Vibrio parahaemolyticus
NAMRU-6_Lambayeque	Lambayeque	24/02/2006	2006	Faecal	Vibrio parahaemolyticus
NAMRU-6_Lambayeque	Lambayeque	09/03/2006	2006	Faecal	Vibrio parahaemolyticus
NAMRU-6_Lambayeque	Lambayeque	15/03/2006	2006	Faecal	Vibrio parahaemolyticus
NAMRU-6_Lambayeque	Lambayeque	15/03/2006	2006	Faecal	Vibrio parahaemolyticus
NAMRU-6_Callao-DAC	Lima	20/04/1998	1998	Faecal	Vibrio parahaemolyticus
NAMRU-6_Callao-DAC	Lima	02/05/1998	1998	Faecal	Vibrio parahaemolyticus
NAMRU-6_Callao-DAC	Lima	24/04/1998	1998	Faecal	Vibrio parahaemolyticus
NAMRU-6_Callao-DAC	Lima	17/04/1998	1998	Faecal	Vibrio parahaemolyticus
NAMRU-6_Callao-DAC	Lima	17/04/1998	1998	Faecal	Vibrio parahaemolyticus
NAMRU-6_Callao-DAC	Lima	23/04/1998	1998	Faecal	Vibrio parahaemolyticus
NAMRU-6_Lima-HospMaternoInfantil	Lima	11/04/1998	1998	Faecal	Vibrio parahaemolyticus
NAMRU-6_Lima-HospFAP	Lima	05/02/1998	1998	Faecal	Vibrio parahaemolyticus
NAMRU-6_Lambayeque	Lambayeque	06/02/2007	2007	Faecal	Vibrio parahaemolyticus
NAMRU-6_Lambayeque	Lambayeque	06/02/2007	2007	Faecal	Vibrio parahaemolyticus
NAMRU-6_Lambayeque	Lambayeque	08/02/2007	2007	Faecal	Vibrio parahaemolyticus
NAMRU-6_Lima-HospFAP	Lima	05/02/1998	1998	Faecal	Vibrio parahaemolyticus
NAMRU-6_Lima-HospFAP	Lima	02/03/1998	1998	Faecal	Vibrio parahaemolyticus
NAMRU-6_Lima-HospFAP	Lima	16/03/1998	1998	Faecal	Vibrio parahaemolyticus
NAMRU-6_Lambayeque	Lambayeque	26/01/2007	2007	Faecal	Vibrio parahaemolyticus
NAMRU-6_Lima-HospFAP	Lima	17/03/1998	1998	Faecal	Vibrio parahaemolyticus
NAMRU-6_-	Lima	15/06/1998	1998	Faecal	Vibrio parahaemolyticus
NAMRU-6_Lima-HospMaternoInfantil	Lima	14/01/2008	2008	Faecal	Vibrio parahaemolyticus
NAMRU-6_Lima-HospMaternoInfantil	Lima	09/01/2008	2008	Faecal	Vibrio parahaemolyticus
NAMRU-6_Lima-EmergenciasPediatricas	Lima	25/01/2008	2008	Faecal	Vibrio parahaemolyticus
NAMRU-6_Lima-Surco	Lima	10/04/2008	2008	Faecal	Vibrio parahaemolyticus
NAMRU-6_Lima-Surco	Lima	16/04/2008	2008	Faecal	Vibrio parahaemolyticus
NAMRU-6_Callao-DAC	Lima	17/04/1998	1998	Faecal	Vibrio parahaemolyticus
NAMRU-6_Callao-DAC	Lima	22/04/1998	1998	Faecal	Vibrio parahaemolyticus
NAMRU-6_-	Lima	25/05/1998	1998	Faecal	Vibrio parahaemolyticus
NAMRU-6_Piura	Piura	02/02/2009	2009	Faecal	Vibrio parahaemolyticus
NAMRU-6_-	Lima	26/03/2009	2009	Faecal	Vibrio parahaemolyticus
NAMRU-6_Lima-EmergenciasPediatricas	Lima	03/05/2009	2009	Faecal	Vibrio parahaemolyticus
NAMRU-6_Callao-DAC	Lima	29/03/1999	1999	Faecal	Vibrio parahaemolyticus
NAMRU-6_Callao-DAC	Lima	29/03/1999	1999	Faecal	Vibrio parahaemolyticus
NAMRU-6_Lima-EmergenciasPediatricas	Lima	19/01/2010	2010	Faecal	Vibrio parahaemolyticus
NAMRU-6_Lima-ISN	Lima	10/02/2010	2010	Faecal	Vibrio parahaemolyticus
NAMRU-6_-	Lima	03/09/1997	1997	Faecal	Vibrio parahaemolyticus
NAMRU-6_Lima-HospMaternoInfantil	Lima	23/03/2011	2011	Faecal	Vibrio parahaemolyticus
NAMRU-6_Lima-HospMaternoInfantil	Lima	23/03/2011	2011	Faecal	Vibrio parahaemolyticus
NAMRU-6_Lima-HospMaternoInfantil	Lima	06/04/2011	2011	Faecal	Vibrio parahaemolyticus
NAMRU-6_Callao-DAC	Lima	25/11/1997	1997	Faecal	Vibrio parahaemolyticus
NAMRU-6_Callao-DAC	Lima	15/12/1997	1997	Faecal	Vibrio parahaemolyticus
NAMRU-6_Callao-DAC	Lima	03/09/1997	1997	Faecal	Vibrio parahaemolyticus
NAMRU-6_Lima-HospMaternoInfantil	Lima	13/04/2000	2000	Faecal	Vibrio parahaemolyticus
NAMRU-6_Lima-HospMaternoInfantil	Lima	16/05/2000	2000	Faecal	Vibrio parahaemolyticus
NAMRU-6_Callao-DAC	Lima	20/12/1997	1997	Faecal	Vibrio parahaemolyticus
NAMRU-6_Lima-HospRebagliati	Lima	02/01/1998	1998	Faecal	Vibrio parahaemolyticus
NAMRU-6_Lima-HospRebagliati	Lima	05/01/1998	1998	Faecal	Vibrio parahaemolyticus
NAMRU-6_Lima-HospRebagliati	Lima	05/01/1998	1998	Faecal	Vibrio parahaemolyticus
NAMRU-6_Lima-HospMaternoInfantil	Lima	27/12/2001	2001	Faecal	Vibrio parahaemolyticus
NAMRU-6_Lima-HospMaternoInfantil	Lima	04/01/2002	2002	Faecal	Vibrio parahaemolyticus
NAMRU-6_Lima-SanBorja	Lima	10/04/2002	2002	Faecal	Vibrio parahaemolyticus
NAMRU-6_Lima-SanBorja	Lima	10/04/2002	2002	Faecal	Vibrio parahaemolyticus
NAMRU-6_Lima-SanBorja	Lima	10/04/2002	2002	Faecal	Vibrio parahaemolyticus
NAMRU-6_Lima-SanBorja	Lima	10/04/2002	2002	Faecal	Vibrio parahaemolyticus
NAMRU-6_Callao-DAC	Lima	26/12/1997	1997	Faecal	Vibrio parahaemolyticus
NAMRU-6_Callao-DAC	Lima	04/01/1998	1998	Faecal	Vibrio parahaemolyticus
NAMRU-6_Callao-DAC	Lima	06/01/1998	1998	Faecal	Vibrio parahaemolyticus
NAMRU-6_Callao-DAC	Lima	29/12/1997	1997	Faecal	Vibrio parahaemolyticus
NAMRU-6_Callao-DAC	Lima	19/01/1998	1998	Faecal	Vibrio parahaemolyticus
NAMRU-6_Callao-DAC	Lima	12/01/1998	1998	Faecal	Vibrio parahaemolyticus
NAMRU-6_Callao-DAC	Lima	12/01/1998	1998	Faecal	Vibrio parahaemolyticus
NAMRU-6_Callao-DAC	Lima	19/01/1998	1998	Faecal	Vibrio parahaemolyticus
NAMRU-6_Callao-DAC	Lima	19/01/1998	1998	Faecal	Vibrio parahaemolyticus
NAMRU-6_Callao-DAC	Lima	12/01/1998	1998	Faecal	Vibrio parahaemolyticus
NAMRU-6_Callao-DAC	Lima	07/01/1998	1998	Faecal	Vibrio parahaemolyticus
NAMRU-6_-	Lima	11/12/1997	1997	Faecal	Vibrio parahaemolyticus
NAMRU-6_Arequipa	Arequipa	17/01/1998	1998	Faecal	Vibrio parahaemolyticus
NAMRU-6_Arequipa	Arequipa	21/10/1997	1997	Faecal	Vibrio parahaemolyticus
NAMRU-6_Arequipa	Arequipa	27/10/1997	1997	Faecal	Vibrio parahaemolyticus
NAMRU-6_Arequipa	Arequipa	28/10/1997	1997	Faecal	Vibrio parahaemolyticus
NAMRU-6_Arequipa	Arequipa	28/10/1997	1997	Faecal	Vibrio parahaemolyticus
NAMRU-6_Arequipa	Arequipa	28/10/1997	1997	Faecal	Vibrio parahaemolyticus
NAMRU-6_Arequipa	Arequipa	17/11/1997	1997	Faecal	Vibrio parahaemolyticus
NAMRU-6_Arequipa	Arequipa	17/11/1997	1997	Faecal	Vibrio parahaemolyticus
NAMRU-6_Callao-DAC	Lima	03/03/1998	1998	Faecal	Vibrio parahaemolyticus
NAMRU-6_Callao-DAC	Lima	06/01/1998	1998	Faecal	Vibrio parahaemolyticus
NAMRU-6_Callao-DAC	Lima	12/01/1998	1998	Faecal	Vibrio parahaemolyticus
NAMRU-6_Callao-DAC	Lima	01/03/1998	1998	Faecal	Vibrio parahaemolyticus
NAMRU-6_Callao-DAC	Lima	07/03/1998	1998	Faecal	Vibrio parahaemolyticus
NAMRU-6_Callao-DAC	Lima	10/03/1998	1998	Faecal	Vibrio parahaemolyticus

NAMRU-6_Callao-DAC	Lima	11/03/1998	1998	Faecal	Vibrio parahaemolyticus
NAMRU-6_-	Lima	02/01/1998	1998	Faecal	Vibrio parahaemolyticus
NAMRU-6_-	Lima	12/01/1998	1998	Faecal	Vibrio parahaemolyticus
NAMRU-6_Lima-HospFAP	Lima	12/01/1998	1998	Faecal	Vibrio parahaemolyticus
NAMRU-6_-	Lima	17/01/1998	1998	Faecal	Vibrio parahaemolyticus
NAMRU-6_Lima-HospMaternoInfantil	Lima	13/02/1998	1998	Faecal	Vibrio parahaemolyticus
NAMRU-6_Ancon	Lima	08/02/1994	1994	Faecal	Vibrio parahaemolyticus
NAMRU-6_Ancon	Lima	08/02/1994	1994	Faecal	Vibrio parahaemolyticus
NAMRU-6_Ancon	Lima	08/02/1994	1994	Faecal	Vibrio parahaemolyticus
NAMRU-6_Ancon	Lima	08/02/1994	1994	Faecal	Vibrio parahaemolyticus
NAMRU-6_Ancon	Lima	08/02/1994	1994	Faecal	Vibrio parahaemolyticus
NAMRU-6_Ancon	Lima	08/02/1994	1994	Faecal	Vibrio parahaemolyticus
NAMRU-6_Ancon	Lima	08/02/1994	1994	Faecal	Vibrio parahaemolyticus
NAMRU-6_Ancon	Lima	08/02/1994	1994	Faecal	Vibrio parahaemolyticus
NAMRU-6_Ancon	Lima	08/02/1994	1994	Faecal	Vibrio parahaemolyticus
NAMRU-6_Ancon	Lima	08/02/1994	1994	Faecal	Vibrio parahaemolyticus
NAMRU-6_Ancon	Lima	08/02/1994	1994	Faecal	Vibrio parahaemolyticus
NAMRU-6_Ancon	Lima	08/02/1994	1994	Faecal	Vibrio parahaemolyticus
NAMRU-6_Ancon	Lima	08/02/1994	1994	Faecal	Vibrio parahaemolyticus
NAMRU-6_Ancon	Lima	08/02/1994	1994	Faecal	Vibrio parahaemolyticus
NAMRU-6_Ancon	Lima	08/02/1994	1994	Faecal	Vibrio parahaemolyticus
NAMRU-6_Ancon	Lima	08/02/1994	1994	Faecal	Vibrio parahaemolyticus
NAMRU-6_Ancon	Lima	08/02/1994	1994	Faecal	Vibrio parahaemolyticus
NAMRU-6_Ancon	Lima	08/02/1994	1994	Faecal	Vibrio parahaemolyticus
NAMRU-6_Ancon	Lima	08/02/1994	1994	Faecal	Vibrio parahaemolyticus
NAMRU-6_Ancon	Lima	08/02/1994	1994	Faecal	Vibrio parahaemolyticus
NAMRU-6_Ancon	Lima	08/02/1994	1994	Faecal	Vibrio parahaemolyticus
NAMRU-6_Ancon	Lima	08/02/1994	1994	Faecal	Vibrio parahaemolyticus
NAMRU-6_Ancon	Lima	08/02/1994	1994	Faecal	Vibrio parahaemolyticus
NAMRU-6_Ancon	Lima	08/02/1994	1994	Faecal	Vibrio parahaemolyticus
NAMRU-6_Ancon	Lima	08/02/1994	1994	Faecal	Vibrio parahaemolyticus
NAMRU-6_Ancon	Lima	08/02/1994	1994	Faecal	Vibrio parahaemolyticus
NAMRU-6_Ancon	Lima	08/02/1994	1994	Faecal	Vibrio parahaemolyticus
NAMRU-6_Ancon	Lima	08/02/1994	1994	Faecal	Vibrio parahaemolyticus
NAMRU-6_Ancon	Lima	08/02/1994	1994	Faecal	Vibrio parahaemolyticus
NAMRU-6_Ancon	Lima	08/02/1994	1994	Faecal	Vibrio parahaemolyticus
NAMRU-6_Ancon	Lima	08/02/1994	1994	Faecal	Vibrio parahaemolyticus
NAMRU-6_Ancon	Lima	08/02/1994	1994	Faecal	Vibrio parahaemolyticus
NAMRU-6_Ancon	Lima	08/02/1994	1994	Faecal	Vibrio parahaemolyticus
NAMRU-6_Ancon	Lima	08/02/1994	1994	Faecal	Vibrio parahaemolyticus
NAMRU-6_Ancon	Lima	08/02/1994	1994	Faecal	Vibrio parahaemolyticus
NAMRU-6_Ancon	Lima	08/02/1994	1994	Faecal	Vibrio parahaemolyticus
NAMRU-6_Ancon	Lima	08/02/1994	1994	Faecal	Vibrio parahaemolyticus
NAMRU-6_Ancon	Lima	08/02/1994	1994	Faecal	Vibrio parahaemolyticus
NAMRU-6_Ancon	Lima	08/02/1994	1994	Faecal	Vibrio parahaemolyticus
NAMRU-6_Ancon	Lima	08/02/1994	1994	Faecal	Vibrio parahaemolyticus
NAMRU-6_Ancon	Lima	08/02/1994	1994	Faecal	Vibrio parahaemolyticus
NAMRU-6_Ancon	Lima	08/02/1994	1994	Faecal	Vibrio parahaemolyticus
NAMRU-6_Ancon	Lima	08/02/1994	1994	Faecal	Vibrio parahaemolyticus
NAMRU-6_Ancon	Lima	08/02/1994	1994	Faecal	Vibrio parahaemolyticus
NAMRU-6_Ancon	Lima	08/02/1994	1994	Faecal	Vibrio parahaemolyticus
NAMRU-6_Ancon	Lima	08/02/1994	1994	Faecal	Vibrio parahaemolyticus
NAMRU-6_Ancon	Lima	08/02/1994	1994	Faecal	Vibrio parahaemolyticus
NAMRU-6_Ancon	Lima	08/02/1994	1994	Faecal	Vibrio parahaemolyticus
NAMRU-6_Ancon	Lima	08/02/1994	1994	Faecal	Vibrio parahaemolyticus
NAMRU-6_Ancon	Lima	08/02/1994	1994	Faecal	Vibrio parahaemolyticus
NAMRU-6_Ancon	Lima	08/02/1994	1994	Faecal	Vibrio parahaemolyticus
NAMRU-6_Ancon	Lima	08/02/1994	1994	Faecal	Vibrio parahaemolyticus
NAMRU-6_Ancon	Lima	08/02/1994	1994	Faecal	Vibrio parahaemolyticus
NAMRU-6_Ancon	Lima	08/02/1994	1994	Faecal	Vibrio parahaemolyticus
NAMRU-6_Ancon	Lima	08/02/1994	1994	Faecal	Vibrio parahaemolyticus
NAMRU-6_Ancon	Lima	08/02/1994	1994	Faecal	Vibrio parahaemolyticus
NAMRU-6_Ancon	Lima	08/02/1994	1994	Faecal	Vibrio parahaemolyticus
NAMRU-6_Ancon	Lima	08/02/1994	1994	Faecal	Vibrio parahaemolyticus
NAMRU-6_Ancon	Lima	08/02/1994	1994	Faecal	Vibrio parahaemolyticus
NAMRU-6_Ancon	Lima	08/02/1994	1994	Faecal	Vibrio parahaemolyticus
NAMRU-6_Ancon	Lima	08/02/1994	1994	Faecal	Vibrio parahaemolyticus
NAMRU-6_Ancon	Lima	08/02/1994	1994	Faecal	Vibrio parahaemolyticus
NAMRU-6_Ancon	Lima	08/02/1994	1994	Faecal	Vibrio parahaemolyticus
NAMRU-6_-	Lima	15/04/1994	1994	Faecal	Vibrio parahaemolyticus
NAMRU-6_-	Lima	15/04/1994	1994	Faecal	Vibrio parahaemolyticus

Supplementary Table S2: Accession numbers for VpST3 sequences used

Strain	BioSample	BioProject	Isolation type	Collection date	Location	Region
091-10	SAMN15428964	PRJNA643807	clinical	2010	Peru	
092-10	SAMN15428968	PRJNA643807	clinical	2010	Peru	
1027-00	SAMN15428943	PRJNA643807	clinical	2000	Peru	
119-10	SAMN15428971	PRJNA643807	clinical	2010	Peru	
1202-11	SAMN12364836	PRJNA556706	clinical	2011	Peru	
1218-11	SAMN15428974	PRJNA643807	clinical	2011	Peru	
1254-07	SAMN15428950	PRJNA643807	clinical	2007	Peru	
1259-07	SAMN15428948	PRJNA643807	clinical	2007	Peru	
1260-07	SAMN15428949	PRJNA643807	clinical	2007	Peru	
164-16	SAMN12364846	PRJNA556706	clinical	2016	Peru	
165-16	SAMN15429002	PRJNA643807	clinical	2016	Peru	
167-16	SAMN15429001	PRJNA643807	clinical	2016	Peru	
202-10	SAMN15428967	PRJNA643807	clinical	2010	Peru	
203-10	SAMN15428963	PRJNA643807	clinical	2010	Peru	
2214-17	SAMN12364848	PRJNA556706	clinical	2017	Peru	
223-10	SAMN15428972	PRJNA643807	clinical	2010	Peru	
243-09	SAMN15428952	PRJNA643807	clinical	2009	Peru	
2434-00	SAMN15428944	PRJNA643807	clinical	2000	Peru	
249-15	SAMN12364841	PRJNA556706	clinical	2015	Peru	
2568-00	SAMN15428945	PRJNA643807	clinical	2000	Peru	
276-15	SAMN12364842	PRJNA556706	clinical	2015	Peru	
277-15	SAMN15428982	PRJNA643807	clinical	2015	Peru	
293-10	SAMN15428966	PRJNA643807	clinical	2010	Peru	
304-10	SAMN15428965	PRJNA643807	clinical	2010	Peru	
325-00	SAMN15428940	PRJNA643807	clinical	2000	Peru	
327-00	SAMN15428942	PRJNA643807	clinical	2000	Peru	
361-10	SAMN15428969	PRJNA643807	clinical	2010	Peru	
403-00	SAMN15428941	PRJNA643807	clinical	2000	Peru	
404-00	SAMN15428938	PRJNA643807	clinical	2000	Peru	
454-10	SAMN15428970	PRJNA643807	clinical	2010	Peru	
686-17	SAMN12364847	PRJNA556706	clinical	2017	Peru	
706-00	SAMN15428939	PRJNA643807	clinical	2000	Peru	
A1078098	SAMN40891081	PRJNA1062747	Clinical	1998	Peru	Lima
A12A1298	SAMN40891083	PRJNA1062747	Clinical	1998	Peru	Lima
A4A497	SAMN40891085	PRJNA1062747	Clinical	2007	Peru	Cajamarca
A5A597	SAMN40891086	PRJNA1062747	Clinical	1997	Peru	Lambayeque
ATC220	SAMN02781334	PRJNA233509	clinical	1998	Chile	Antofagasta
B6B699	SAMN40891643	PRJNA1062747	Clinical	1999	Peru	Lambayeque
C6C61	SAMN40891645	PRJNA1062747	Clinical	2001	Peru	Lima
C9C91	SAMN40891646	PRJNA1062747	Clinical	2001	Peru	Iquitos
CEFAS0023	SAMN12254049	PRJNA438219	Clinical (travel)	2016	Colombia	
CFSAN018757	SAMN03941065	PRJNA245882	clinical	1997	Peru	
CFSAN023554	SAMN06077005	PRJNA245882	environmental/other	2005	Chile	
CFSAN023556	SAMN06077007	PRJNA245882	environmental/other	2005	Chile	
CFSAN029652	SAMN08225458	PRJNA245882	environmental/other	2009	Peru	
CFSAN029655	SAMN08225457	PRJNA245882	environmental/other	2009	Peru	
CFSAN029657	SAMN08225459	PRJNA245882	environmental/other	2009	Peru	
D10D103	SAMN40891647	PRJNA1062747	Clinical	2003	Peru	Cajamarca
D3D32	SAMN40891105	PRJNA1062747	Clinical	2002	Peru	Lima
G1	SAMN12364839	PRJNA556706	clinical	2014	Peru	
G6	SAMN15428983	PRJNA643807	clinical	2015	Peru	Lima
G8	SAMN15429003	PRJNA643807	clinical	2016	Peru	Lima
H11	SAMN15428984	PRJNA643807	clinical	2015	Peru	Lima
H12	SAMN15429004	PRJNA643807	clinical	2016	Peru	Lima
Peru-288	SAMN01923801	PRJNA176642	clinical	2001	Peru	
PMA37.5	SAMN05858273	PRJNA345099	clinical	2005-01	Chile	
PMC14.7	SAMN02781336	PRJNA233509	clinical	2007	Chile	PuertoMontt
PMC48	SAMN02781337	PRJNA233509	clinical	2004	Chile	PuertoMontt
PMC58.5	SAMN02781338	PRJNA233509	clinical	2005	Chile	PuertoMontt
PMC58.7	SAMN02781339	PRJNA233509	clinical	2007	Chile	PuertoMontt
PV170	SAMN20804964	PRJNA754786	clinical	2019-05-06	Colombia	Cordoba
PV173	SAMN20804965	PRJNA754787	NA	2019-07-17	Colombia	Cordoba
PV278	SAMN20804967	PRJNA754787	clinical	2019-07-30	Colombia	Bogota
PV280	SAMN20804968	PRJNA754787	clinical	2019-07-24	Colombia	Cordoba
PV53	SAMN20804969	PRJNA754788	clinical	2018-07-30	Colombia	Cordoba
PV85	SAMN20804970	PRJNA754789	clinical	2017-10-16	Colombia	Cordoba
